# Integrated multi-omics analysis reveals hormonal and nutrient networks regulating sugarcane tillering

**DOI:** 10.3389/fpls.2026.1755625

**Published:** 2026-04-20

**Authors:** Shimiao Chen, Bin Shan, Zeping Wang, Yi Song, Yuyan Qin, Li Liu, Qian Qin, Xiang Li, Qinyu Lu

**Affiliations:** 1 Key Laboratory of Guangxi Subtropical Crops Research Institute, Nanning, China; 2 Key Laboratory of Quality and Safety Control for Subtropical Fruit and Vegetable, Ministry of Agriculture and Rural Affairs, Nanning, China; 3 Guangxi Academy of Agricultural Science, Nanning, China; 4 Guangxi Baise Modern Agriculture Technology Research and Extension Center, Management Committee of Baise National Agricultural Science and Technology Zone of Guangxi, Baise, China

**Keywords:** growth–defense trade-off, multi-omics, sugarcane, tillering, WGCNA

## Abstract

**Introduction:**

Sugarcane tillering represents a complex developmental process shaped by multiple interacting molecular networks. To outline a multi-omics regulatory framework linking hormonal, ionomic, and transcriptomic variation to contrasting tillering capacities in sugarcane, we analyzed contrasting genotypes using an integrated multi-omics approach.

**Methods:**

Four field-grown sugarcane genotypes at the peak tillering stage were analyzed using integrated transcriptomics, targeted phytohormone metabolomics, and ionomics, with three biological replicates per genotype × tissue combination.

**Results:**

High-tillering plants maintained a transcriptional and metabolic state optimized for growth, including enhanced expression of photosynthetic and carbon metabolic pathways, marked accumulation of the auxin-associated metabolite tryptamine (Log2FC = 4.97), elevated auxin- and cytokinin-associated metabolites, and preferential enrichment of micronutrients in tiller tissues. In contrast, low-tillering genotypes adopted a stress-prepared phenotype characterized by activation of defense and protein synthesis pathways, reduced 1-aminocyclopropane-1-carboxylic acid (ACC) levels in high-tillering plants relative to low-tillering plants (Log2FC = −2.26), accumulation of jasmonate-, salicylic acid-, and abscisic acid (ABA)-associated metabolites, and distinct ionomic signatures associated with nutrient imbalance. Weighted Gene Co-expression Network Analysis (WGCNA) further revealed a polarized regulatory structure, with the growth-associated module positively correlated with cis-Zeatin riboside (cZR) (yellow-labelled, r = 0.71), whereas the defense-associated module showed strong negative correlations with cZR/cis-Zeatin (cZ) (brown-labelled, r = −0.74/−0.75), and another defense-associated module showed positive correlations with ACC and Salicylic acid (SA) (turquoise-labelled, r = 0.77 and 0.79, respectively) .

**Discussion:**

This study provides an integrated multi-omics framework for understanding sugarcane tillering and highlights a coordinated growth–defense trade-off associated with contrasting tillering strategies.

## Introduction

Sugarcane (*Saccharum* spp. hybrid) is one of the world’s most important cash crops, cultivated extensively across tropical and subtropical regions. Globally, sugarcane contributes over 80% of sugar output and, together with sugar beet (Dinesh Babu et al., 2022), provides about 40% of the feedstock used for bioethanol production (Bušić et al., 2018). Beyond sugar and ethanol, the sugarcane industry is a cornerstone of the modern bio-economy, utilizing by-products such as bagasse for electricity cogeneration and molasses for producing biochemicals and spirits. This industrial versatility confers immense economic and environmental importance. Nevertheless, the global sugarcane industry faces multiple challenges, including rising production costs, pest and disease pressure, and the sustainable management of soil and water resources (Meena et al., 2022).

In China, sugarcane is also a major sugar crop, with its cultivation area and yield accounting for approximately 85–91% of the country’s total sugar production (Li et al., 2024). However, China’s sugarcane industry continues to struggle with high production costs, outdated varieties, and increasing disease pressure (Bai et al., 2019; Chen and Zhao, 2019). Therefore, breeding programs worldwide are focusing on developing high-yielding, high-sugar, disease-resistant, and broadly adaptable varieties to sustain global sugar and bioenergy supplies.

High tillering is a crucial agronomic trait in sugarcane breeding because enhancing yield is the primary objective (Khonghintaisong et al., 2023). High tillering increases stalk density and canopy photosynthetic capacity, enhancing biomass and sugar yield. Thus, high tillering is a key determinant of yield per unit area and an indicator of cultivation efficiency. Additionally, high-tillering varieties may exhibit improved nutrient uptake, particularly in phosphorus-limited soils, and may utilize soil nutrients more efficiently through rhizosphere microbial interactions (Gokul et al., 2023). Under stress conditions such as waterlogging, moderate tillering improves survival, and genotypes with stable tiller development enhance field resilience (Kumar et al., 2015).

However, excessive tillering can impair sugarcane performance. Excessive tiller initiation produces weak or late-emerging shoots that act as metabolic sinks, diverting assimilates from primary culms. Overcrowded tillers intensify competition for nutrients, water, and light during elongation, reducing growth rate, stalk diameter, and sucrose accumulation. Canopy densification restricts ventilation and promotes a humid microenvironment favorable for disease development. Greater lodging risk further reduces mechanical harvest efficiency and depresses juice quality (Li, 2019, 2019; Chaulagain et al., 2020). Therefore, optimizing the number of productive tillers, rather than maximizing tiller initiation, is essential for yield improvement.

Optimizing sugarcane tillering requires integrating breeding strategies and field management practices. Agronomic measures are the first line of control; for example, planting density determines light availability for basal buds, while optimized nutrient and water management ensure that emerging tillers survive to become productive stalks (Silva et al., 2022). Hybrid breeding techniques enable the selection of genotypes with optimal tillering and yield potential. At the molecular level, significant advances have been made. Quantitative trait locus (QTL) mapping has identified loci such as qPCTR-R9, qPCTR-Y28, and qPCTR-Y60, with qPCTR-Y8-1/qRSR-Y8 showing stable expression across environments (Wang et al., 2023). Functional studies in model grasses such as rice and sorghum have delineated conserved tillering regulators, notably *TB1* (TEOSINTE BRANCHED1) (Takeda et al., 2003), *MOC1* (MONOCULM1) (Li et al., 2003), and the strigolactone signaling components (*MAX/D* genes) (Arite et al., 2009). Although homologous genes have been identified in sugarcane, whether they operate through regulatory networks fully comparable to those described in rice and sorghum remains uncertain, given the highly polyploid and heterozygous nature of the sugarcane genome. Gene families such as *ScDIR*, *D27* from Saccharum spontaneum, and *Dof* transcription factors have also been implicated in regulating tillering and stress responses (Cai et al., 2020; Huang et al., 2020). Under drought conditions, genes including LSG1-2, ERF1-2, and SHKA play critical roles in tiller regulation (Tang et al., 2023). Collectively, these findings suggest that sugarcane may share some tillering-related regulatory components with other grasses; however, how these components are functionally integrated within its complex polyploid genome remains poorly understood.

Plant hormones play multifaceted roles in regulating the number, growth, and quality of sugarcane tillers. Hormonal signals mediate intricate crosstalk between growth-promoting and growth-inhibiting pathways, fine-tuning bud outgrowth and tiller survival. Major regulators include indole-3-acetic acid (IAA), gibberellins (GA), ethylene (ETH), cytokinins (CTK), ABA, brassinosteroids (BR), and strigolactones (SLs) (Duan et al., 2019; Patil et al., 2022). Cytokinins synthesized in roots promote axillary bud activation, whereas auxin from the shoot apex enforces apical dominance and inhibits bud outgrowth. Strigolactones, synthesized in response to nutrient cues, repress bud activation by modulating auxin transport and stimulating cytokinin degradation. The hormonal balance is further influenced by nutrient status—for example, nitrogen enrichment promotes cytokinin synthesis while suppressing strigolactone production, strongly favoring tillering (Qi et al., 2025). GA and ethylene also stimulate tiller emergence and elongation. Understanding these hormonal networks provides a foundation for fine-tuning tillering to suit diverse production environments.

Mineral elements play equally critical roles, influencing tillering both directly and via hormone metabolism. Nitrogen (N) strongly affects tiller initiation by supporting chlorophyll, amino acid, and cytokinin biosynthesis (de Oliveira Junior et al., 2023). Phosphorus (P) deficiency severely limits bud outgrowth because it is required for ATP-driven cell division, whereas adequate P supply enhances tiller survival by improving carbohydrate partitioning (Xu et al., 2017a). Potassium (K) maintains enzyme activation and turgor regulation, which are essential for culm elongation, while zinc (Zn) acts as a cofactor in carbohydrate metabolism and auxin biosynthesis (Dhaliwal et al., 2022). Calcium (Ca) strengthens stalk structure and promotes root growth, indirectly supporting tiller formation. However, nutrient antagonism can occur; excessive K or Na may inhibit Ca uptake, reducing tiller number (Costa et al., 2021). Balancing these elements is thus central to the sustainable intensification of global sugarcane systems.

Despite extensive research into individual regulatory factors, the integrated network connecting genetic, hormonal, and nutritional control of sugarcane tillering remains largely unresolved, particularly with respect to how these regulatory layers jointly contribute to contrasting tillering capacities across different tissues in sugarcane. The crop’s highly polyploid and heterozygous genome poses additional challenges, hindering the direct transfer of insights from diploid model species. To address this gap, we integrated transcriptomic, targeted phytohormone metabolomic, and ionomic analyses across main stem and tiller tissues in sugarcane varieties with contrasting tillering capacities, aiming to resolve tissue-dependent regulatory differences and identify key modules associated with tillering. We hypothesized that contrasting tillering phenotypes are associated with differences in tissue-dependent regulatory specialization between source and sink organs. Accordingly, our objective was to identify key modules, hub genes, and multi-omic signatures associated with the systems-level regulation of tillering. By integrating these three omics layers with tissue-resolved sampling across genotypes with contrasting tillering capacities, this work aims to provide new mechanistic insights into the systems-level regulation of sugarcane tillering that cannot be readily obtained from single-omics approaches, offering a theoretical foundation for globally relevant strategies in sugarcane breeding and sustainable cultivation.

## Materials and methods

Four sugarcane varieties exhibiting strongly contrasting tillering capacities were selected from a field experiment conducted at the experimental station of the Qinzhou Branch of Guangxi Academy of Agricultural Sciences, Guangxi, China. The selected varieties represented the strongest tillering contrast among the materials grown in the same field under shared management, with mean tiller numbers (mean ± SE) of 7.83 ± 0.37 in GT07168 (MA), 6.83 ± 0.50 in GL05136 (MB), 4.17 ± 0.64 in GT60 (SA), and 3.83 ± 0.28 in ZZ6 (SB), corresponding to an approximately twofold difference between the high- and low-tillering groups. Each variety was planted in an area of approximately 1 mu under newly planted cane conditions. Samples were collected in May during the peak tillering stage, when tiller emergence had largely stabilized. After excluding border rows, plants were randomly sampled using a plum-blossom sampling pattern. For each genotype, leaves from the main stem (denoted as M) and tiller buds (denoted as T) were collected. Three independent biological replicates were collected per genotype and tissue combination (n=3) to ensure statistical reliability and reproducibility. To capture systemic regulatory signals and local developmental responses associated with tillering, samples were collected from two biologically complementary tissues. Main stem leaves represent the primary source of photosynthates and long-distance hormonal signals (notably auxin) that maintain apical dominance and regulate axillary bud fate. In contrast, tiller buds/stems serve as the local site where bud activation or dormancy is executed. Sampling both tissues, therefore, enables discrimination between systemic regulatory inputs and localized transcriptional and metabolic responses underlying tiller development. Each sample was then divided into two aliquots: one was immediately flash-frozen in liquid nitrogen and stored at –80 °C for subsequent molecular analyses, while the other was fixed at 105 °C and then dried at 65 °C to constant weight for physicochemical analyses.

### Transcriptome sequencing and analysis

Total RNA was isolated from frozen sugarcane samples using TRIzol reagent (Sangon, China), and its quality was confirmed using an Agilent 2100 Bioanalyzer (RIN ≥ 7.8). Paired-end transcriptome sequencing was performed on an Illumina HiSeq 2500 platform (USA) by Sangon Biotech (Shanghai, China). The resulting raw reads were processed using Trimmomatic (v0.36) (Bolger et al., 2014) with the following steps: 1) adapter sequences were removed; 2) reads containing N bases were discarded; 3) low-quality bases from both the 3’ and 5’ ends (Phred score < 20) were trimmed; 4) a 5 bp sliding window was used to trim reads where the average quality dropped below 20; and 5) reads shorter than 35 nt after trimming were discarded. This process yielded high-quality clean reads for assembly.

Given the highly polyploid and heterozygous nature of the sugarcane genome, which poses significant challenges for reference-based read alignment, a high proportion of multi-mapping reads was expected. To address this challenge, we employed a reference-free strategy. First, a *de novo* assembly was performed using the Trinity pipeline (v2.4.0) (Grabherr et al., 2011) with the parameter min_kmer_cov 2 to reconstruct the transcriptome directly from the clean reads. This reference-free approach allows for the reconstruction of transcripts specific to our cultivars, including unique alleles and isoforms.

The completeness of the final assembly was then assessed using BUSCO (v6) (Simão et al., 2015). The analysis was performed in transcriptome mode (-m transcriptome) against the Poales odb12 lineage dataset (n=6,282). To evaluate the assembly’s representativeness, clean reads were mapped back to the assembled unigenes using Bowtie2 (v2.3.2), and the resulting mapping rates were assessed.

To quantify expression levels while explicitly accounting for the expected high proportion of ambiguous reads, gene expression levels were quantified as Transcripts Per Million (TPM) using Salmon (v0.8.2). The Salmon software was explicitly chosen because its quasi-mapping algorithm and underlying statistical model (Expectation-Maximization) probabilistically assign ambiguous reads, thereby providing more accurate transcript abundance estimates than conventional read-counting methods.

A raw read count matrix was also generated by Salmon, which served as the direct input for differential expression analysis using the DESeq2 R package (v1.12.4) (Love et al., 2014). DESeq2 performs internal normalization (the median of ratios) and uses a Wald test for statistical significance. Transcripts with an adjusted p-value (padj) ≤ 0.01 and an absolute log2(Fold Change) ≥ 1 were defined as significantly differentially expressed, using a relatively stringent threshold to reduce false-positive signals in downstream enrichment and integrative analyses. For visualization and co-expression network analysis, expression values were calculated as TPM.

Functional annotation and classification of transcripts were performed using Blast2GO (Conesa et al., 2005), with KEGG pathway annotation supplemented by the KEGG Automatic Annotation Server (KAAS). To investigate the biological functions of the differentially expressed genes, KEGG pathway enrichment analysis was conducted using the R package clusterProfiler (Yu, 2024). The background gene set for this analysis comprised all unigenes that were successfully assigned a KO identifier. P-values were adjusted for multiple testing using the Benjamini-Hochberg (BH) method, and a pathway was considered significantly enriched if the q-value was less than 0.05. RNA-seq results were further validated using quantitative real-time PCR (qRT-PCR) on a CFX Opus Real-Time PCR System (Bio-Rad, USA), with expression levels normalized to actin and analyzed via the 2^-ΔΔCt^ method (Livak and Schmittgen, 2001). Each qRT-PCR experiment was performed in triplicate.

### Targeted phytohormone metabolomics

The phytohormone content analysis was conducted by MetWare Biotechnology Co., Ltd. (Wuhan, China) using an LC-MS/MS platform. Frozen samples (approximately 50 mg) were cryogenically ground and extracted with 1 mL of methanol/water/formic acid solution (15:4:1, v/v/v) containing a mixture of isotope-labeled internal standards, as detailed in **Supplementary Table 1**. The mixture was vortexed, centrifuged, evaporated to dryness, reconstituted in 100 μL of 80% methanol, and filtered through a 0.22 μm filter (Li et al., 2016).

The sample extracts were analyzed using a UHPLC-ESI-MS/MS system (UPLC, ExionLC AD Series; MS, QTRAP 6500+, AB Sciex, USA). Chromatographic separation was performed on a Waters ACQUITY UPLC HSS T3 C18 column (1.8 μm, 2.1 mm × 100 mm) with a mobile phase consisting of water with 0.04% acetic acid (A) and acetonitrile with 0.04% acetic acid (B). The gradient program was as follows: 0–1 min, 5% B; 1–8 min, linear gradient to 95% B; 8–9 min, 95% B; 9.1–12 min, ramped back to 5% B. The flow rate was 0.35 mL/min, and the column temperature was 40 °C (Cai et al., 2014). The mass spectrometer was operated in scheduled multiple reaction monitoring (MRM) mode (Pan et al., 2010), with detailed analytical parameters for the targeted phytohormones and internal standards listed in **Supplementary Table 1**.

For statistical analysis, the quantified phytohormone data were first filtered to exclude compounds with zero values in more than 50% of all samples. For the retained variables, zero or missing values were imputed using half of the minimum non-zero value of the corresponding compound. The processed data were then subjected to unit variance (UV) scaling so that compounds with different absolute abundances and variances contributed more comparably to downstream multivariate analyses. To provide an initial overview of hormonal variation across genotypes and tissues, a heatmap with hierarchical clustering (Euclidean distance, Ward’s D2 method) was constructed using the mean values of biological replicates. Representative hormones from each class (ABA, auxin, cytokinin, ethylene, gibberellin, jasmonate, and salicylic acid) were shown in the main text, whereas the complete heatmap was provided as supplementary material. To evaluate global hormonal variation and identify discriminative phytohormones, the scaled data were subjected to multivariate statistical analyses (detailed in the subsequent section). Specifically, differential metabolites associated with tillering capacity were screened based on a variable importance in the projection (VIP) score > 1 derived from the OPLS-DA model.

### Ionomic analysis

The ionomics analysis was performed following the protocol described by Xiao et al. (2021). For each sample, approximately 0.5 g of crushed, dried material was weighed and subsequently digested in a mixture of 9 mL of nitric acid (HNO_3_) and 2 mL of hydrogen peroxide (H_2_O_2_) using a microwave digestion system (MARS 6, CEM, USA). The digestion program was set to 190 °C for 30 minutes. Following digestion, the samples were cooled, diluted to a final volume of 50 mL with deionized water, and filtered through a 0.45 µm filter. The filtrates were stored at 4 °C prior to analysis. The concentrations of 29 elements (Aluminum, Al; Arsenic, As; Boron, B; Barium, Ba; Calcium, Ca; Cadmium, Cd; Cobalt, Co; Chromium, Cr; Copper, Cu; Iron, Fe; Potassium, K; Lithium, Li; Magnesium, Mg; Manganese, Mn; Molybdenum, Mo; Sodium, Na; Nickel, Ni; Phosphorus, P; Lead, Pb; Palladium, Pd; Antimony, Sb; Selenium, Se; Silicon, Si; Tin, Sn; Strontium, Sr; Titanium, Ti; Vanadium, V; Tungsten, W; and Zinc, Zn) were quantified using an Inductively Coupled Plasma Optical Emission Spectrometer (EXPEC 6500, EXPEC Technology, China).

For ionomic data analysis, the raw concentration data were UV-scaled (unit variance scaling) by subtracting the mean and dividing by the standard deviation for each element. The scaled data were visualized using a heatmap with hierarchical clustering (Euclidean distance, Ward’s D2 method) to provide an overview of the elemental profiles across genotypes and tissues. Group means of biological replicates were used for heatmap construction. For the main-text figure, 20 representative elements were selected across three nutritional categories—macronutrients, micronutrients, and non-essential elements—while the complete heatmap of all 29 elements was provided as supplementary material. Global elemental variation and discriminative ions were subsequently evaluated through multivariate statistical analyses (described below). Differential elements between contrasting tillering genotypes were identified based on a variable importance in the projection (VIP) score > 1 from the corresponding OPLS-DA model, with fold changes calculated to quantify the magnitude of differences.

### Integrated multi-omics and network analysis

To integrate the multi-omics datasets and identify key regulatory networks, WGCNA was performed. Prior to network construction, the expression matrix was rigorously filtered to ensure data quality; specifically, only genes with a TPM > 1 in at least 75% of the samples were retained, and those exhibiting zero variance across all samples were removed. The resulting filtered, log-transformed TPM matrix was utilized to construct the co-expression network using the WGCNA R package (Langfelder and Horvath, 2008), while quantitative data from metabolomics and ionomics were integrated as external traits to identify trait-associated gene modules. A soft-thresholding power (β) of 12 was selected as it represented the lowest power satisfying the scale-free topology criterion (**Supplementary Figures S1**, **S2**). The associations between module eigengenes and individual traits (hormone and mineral levels) were quantified using Pearson correlation coefficients, and the resulting p values were further adjusted for multiple comparisons using the Benjamini–Hochberg false discovery rate (FDR) procedure, implemented via the p.adjust function in R (R Core Team 2024), to account for the number of module–trait pairs tested simultaneously. Associations with FDR < 0.05 were considered statistically significant, while those with raw p < 0.05 but not surviving FDR correction were retained as exploratory patterns for hypothesis generation. To elucidate the biological functions of these modules, KEGG pathway enrichment analysis was performed on the genes within each significant module using the R package clusterProfiler (Yu, 2024), based on previously obtained KO annotations, with a q-value threshold of < 0.05. Finally, hub genes within critical modules were identified using a two-step approach (Langfelder and Horvath, 2008): first, filtering genes based on minimum statistical significance (p_GS < 0.05) and high module membership (kME > 0.8), followed by ranking the filtered candidates based on a combined score (Score = kME * |GS|). This use of kME and GS thresholds is a standard method for establishing network centrality and identifying genes with potential vital roles in the trait (Yang et al., 2021). The top 20-ranked genes for each significant module-trait association were selected for detailed analysis.

### Multivariate statistical analysis

To evaluate the overall variance and sample clustering patterns across the three omics datasets, multivariate statistical analyses were performed. For the targeted phytohormone and ionomic data, unit variance (UV) scaling was applied to the raw quantitative data, and principal component analysis (PCA) together with orthogonal partial least squares-discriminant analysis (OPLS-DA) were performed in R. PCA was used as an unsupervised dimensionality-reduction approach to summarize the dominant axes of variation and to facilitate visualization of genotype- and tissue-associated separation patterns; it also allowed assessment of the relative coherence of biological replicates within each group. For the transcriptomic data, a Euclidean distance matrix was calculated from log2-transformed TPM values, followed by Principal Coordinate Analysis (PCoA) using the pcoa function in the R package ape.

Furthermore, to statistically validate the sample clustering patterns and quantify the variance explained by experimental factors across all three omics layers, Permutational Multivariate Analysis of Variance (PERMANOVA) was employed. Global and two-way PERMANOVA were conducted using the adonis2 function in the R package vegan with 999 permutations, based on Euclidean distance matrices for transcriptomic (log2-transformed TPM), phytohormone (UV-scaled), and ionomic (UV-scaled) profiles. To robustly satisfy the statistical assumptions of PERMANOVA, the homogeneity of multivariate dispersions among experimental groups was confirmed prior to interpretation using the betadisper and permutest functions within the same package.

## Results

### Analysis of transcriptomic data

The quality and completeness of the *de novo*-assembled transcriptome were rigorously assessed first. The final assembly yielded 361,737 unigenes with an N50 of 634 bp. To evaluate the completeness of the gene content, BUSCO (v6.0.0) was used to assess transcriptome completeness under the euk_trans mode with the Poales lineage dataset (poales_odb12, released 01 Jul 2025; 6,282 BUSCOs), which revealed a high completeness score of 77.7%. This completeness score is comparable to or exceeds those reported in tissue-specific *de novo* transcriptomes of sugarcane and other Poales species (Waterhouse et al., 2018; Souza et al., 2019; Li et al., 2023). To further validate how well the assembly represented the raw sequencing data, clean reads were mapped back to the assembled unigenes, yielding overall mapping rates of 75.56%-79.43% across all samples. Notably, the mapping results showed a high proportion of multi-mapping reads (23.18% to 26.78%) and a lower proportion of uniquely mapping reads (42.38% to 42.65%), a typical characteristic of transcriptomic data from a complex, heterozygous polyploid organism.

Finally, to confirm the accuracy of the expression levels quantified from this assembly, a set of representative differentially expressed genes was selected for quantitative real-time PCR (qRT-PCR) analysis. The qRT-PCR expression patterns were highly consistent with the transcriptomic data, confirming the overall reliability of our sequencing results (**Supplementary Table 2**).

Having established a robust, validated transcriptomic dataset, we tested our primary hypothesis that the interaction between tillering capacity and tissue type drives transcriptomic variation. Principal Coordinate Analysis (PCoA) was performed on the Euclidean distance matrix derived from the log2(TPM + 1)-transformed and UV-scaled transcript abundance matrix from multi-tillering (MA, MB) and low-tillering (SA, SB) sugarcane varieties, analyzing tissues from both main stems (M) and tiller stems (T). The PCoA was highly effective, with the first three principal coordinates capturing the majority of total transcriptomic variance (PCo1: 61.36%, PCo2: 12.42%, and PCo3: 10.53%), collectively explaining 84.31% of the dataset’s variation. Global PERMANOVA confirmed that the eight sample groups explained a statistically significant proportion of transcriptomic variance (R² = 0.437, F = 1.78, p = 0.001), with homogeneous within-group dispersions verified prior to interpretation (Beta-dispersion test, p = 0.679).

**Figure 1:**
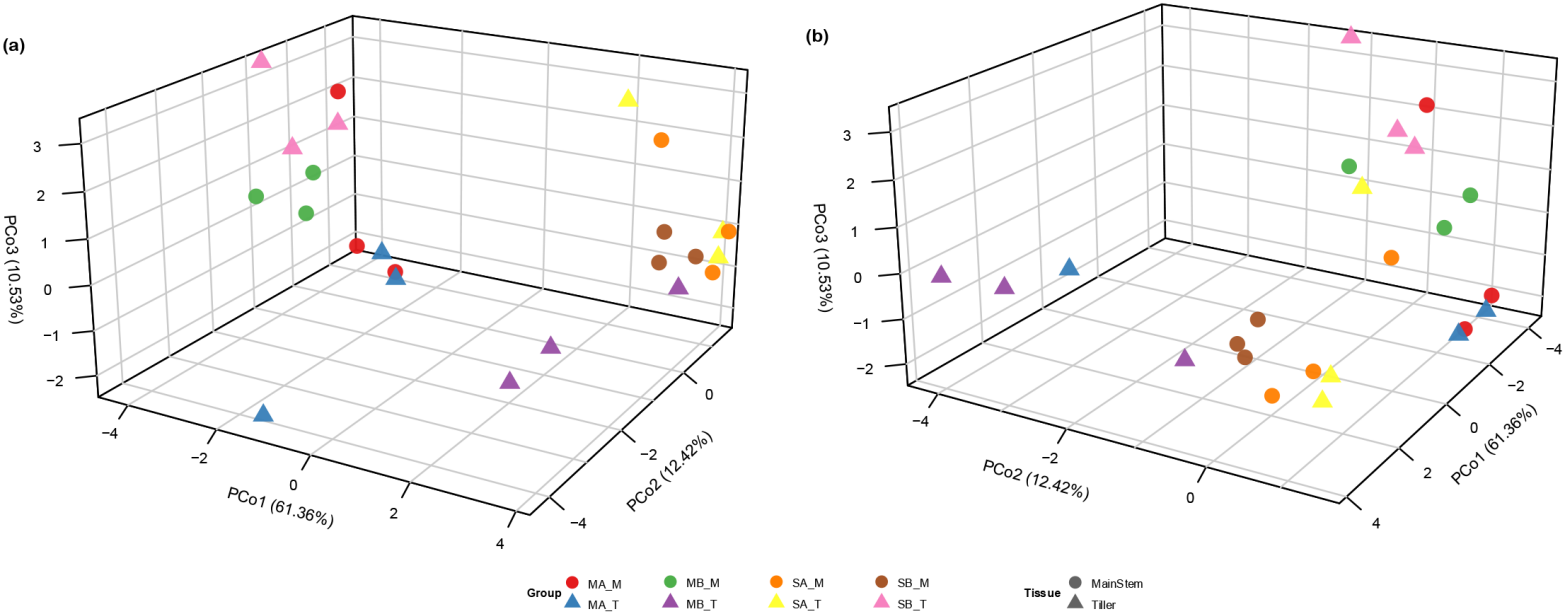
Three-dimensional PCoA of transcriptome profiles across tissues and tillering genotypes. Principal Coordinate Analysis (PCoA) of transcriptome profiles from main stem (M) and tiller stem (T) tissues of high-tillering (MA, MB) and low-tillering (SA, SB) genotypes (n = 3 biological replicates per group). The first three principal coordinates collectively explained 84.3% of total variance (PCo1: 61.4%, PCo2: 12.4%, PCo3: 10.5%). Two viewing angles of the same three-dimensional ordination are presented: **(a)** PCo1-forward view and **(b)** PCo2-forward view.

The score plot (**Figure 1**) revealed that tillering capacity was the primary determinant of transcriptomic architecture. High-tillering genotypes (MA, MB; mean PCo1 = −1.71) and low-tillering genotypes (SA, SB; mean PCo1 = +1.71) were clearly separated along PCo1, indicating fundamentally different genome-wide expression programs associated with this trait. Two-way PERMANOVA confirmed tillering capacity as a significant driver of transcriptomic variation (R² = 0.082, F = 2.10, p = 0.001). Critically, the interaction term between tillering capacity and tissue type was also statistically significant (R² = 0.082, F = 2.10, p = 0.001), providing formal statistical support for tissue-type effects being dependent on tillering phenotype.

This interaction is clearly reflected in the spatial structure of the PCoA plot: when high-tillering varieties (MA and MB combined) are considered together, the centroid distance between their main stem and tiller stem samples (5.213) is markedly greater than the corresponding distance in low-tillering varieties (SA and SB combined; 4.193), representing a 1.24-fold difference. This greater transcriptomic divergence between stem compartments in high-tillering genotypes suggests that a high-tillering phenotype is supported by a more dynamic and specialized transcriptional reprogramming between the source (main stem) and sink (tiller) tissues. In contrast, low-tillering varieties exhibit a more conservative and less differentiated expression profile across tissues.

Taken together, the PCoA and PERMANOVA analyses establish tillering capacity as the dominant factor structuring the sugarcane transcriptome (R² = 0.082, p = 0.001), with tissue-specific transcriptional divergence being significantly more pronounced in high-tillering genotypes — a pattern fully consistent with the greater source–sink developmental complexity underlying their superior tillering potential. KEGG pathway enrichment analysis (**Supplementary Figure S3–1**, **S3-2**) revealed pronounced differences in biological processes between high-tillering and low-tillering sugarcane varieties and between the main stem and tiller tissues. High-tillering varieties showed a broad spectrum of significantly enriched pathways across tissues (121 pathways), reflecting active and diverse metabolic regulation, whereas low-tillering varieties showed only limited enrichment (9 pathways). When directly comparing high- and low-tillering genotypes within the same tissue type, 63 and 142 pathways were enriched in the main stem and tiller comparisons, indicating extensive reprogramming of metabolic and signaling networks in high-tillering backgrounds.

KEGG pathway analysis of DEGs revealed clear functional distinctions between high- and low-tillering genotypes, particularly in the main stem (M) tissues, which represent the site of tillering potential (**Supplementary Table 3**).

High-tillering main stems (MA_M and MB_M) were significantly enriched (UP-regulated) for pathways related to high anabolic and energetic activity. These included Photosynthesis - antenna proteins (ko00196), Photosynthesis (ko00195), Carbon fixation in photosynthetic organisms (ko00710), Starch and sucrose metabolism (ko00500), and Nitrogen metabolism (ko00910). Notably, these high-tillering stems also showed strong enrichment for key secondary metabolism pathways, specifically Phenylpropanoid biosynthesis (ko00940) and Flavonoid biosynthesis (ko00941).

In stark contrast, the low-tillering main stems (SA_M and SB_M) showed a completely different profile. They were strongly enriched (DOWN-regulated in the MA_M vs SA_M and MB_M vs SB_M comparisons) for pathways related to protein production machinery: Ribosome (ko03010), Ribosome biogenesis in eukaryotes (ko03008), and Aminoacyl-tRNA biosynthesis (ko00970). Notably, this strong enrichment of protein synthesis machinery was observed despite the low-tillering, low-growth phenotype of these genotypes, suggesting a complex relationship between translational pathway activation and productive growth. These data identify translation-related enrichment as a characteristic molecular feature of low-tillering main stems, but the functional significance of this pattern requires cautious interpretation.

A different functional pattern emerged when comparing the tiller (T) tissues. In the MA_T vs SA_T and MA_T vs SB_T comparisons, it was the low-tillering tillers (SA_T, SB_T) that showed significant enrichment (DOWN-regulated) for defense- and stress-related pathways, including Plant-pathogen interaction (ko04626) and MAPK signaling pathway - plant (ko04016).

Together, these findings indicate a key functional trade-off: high-tillering genotypes are distinguished by main stems primed for high-energy production (Photosynthesis, carbon metabolism) and secondary metabolism (phenylpropanoids), while low-tillering genotypes exhibit main stems curiously enriched for protein synthesis machinery and tillers that appear to be under significant stress (defense pathway activation).

### Analysis of hormone-targeted metabolomics data

**Figure 2:**
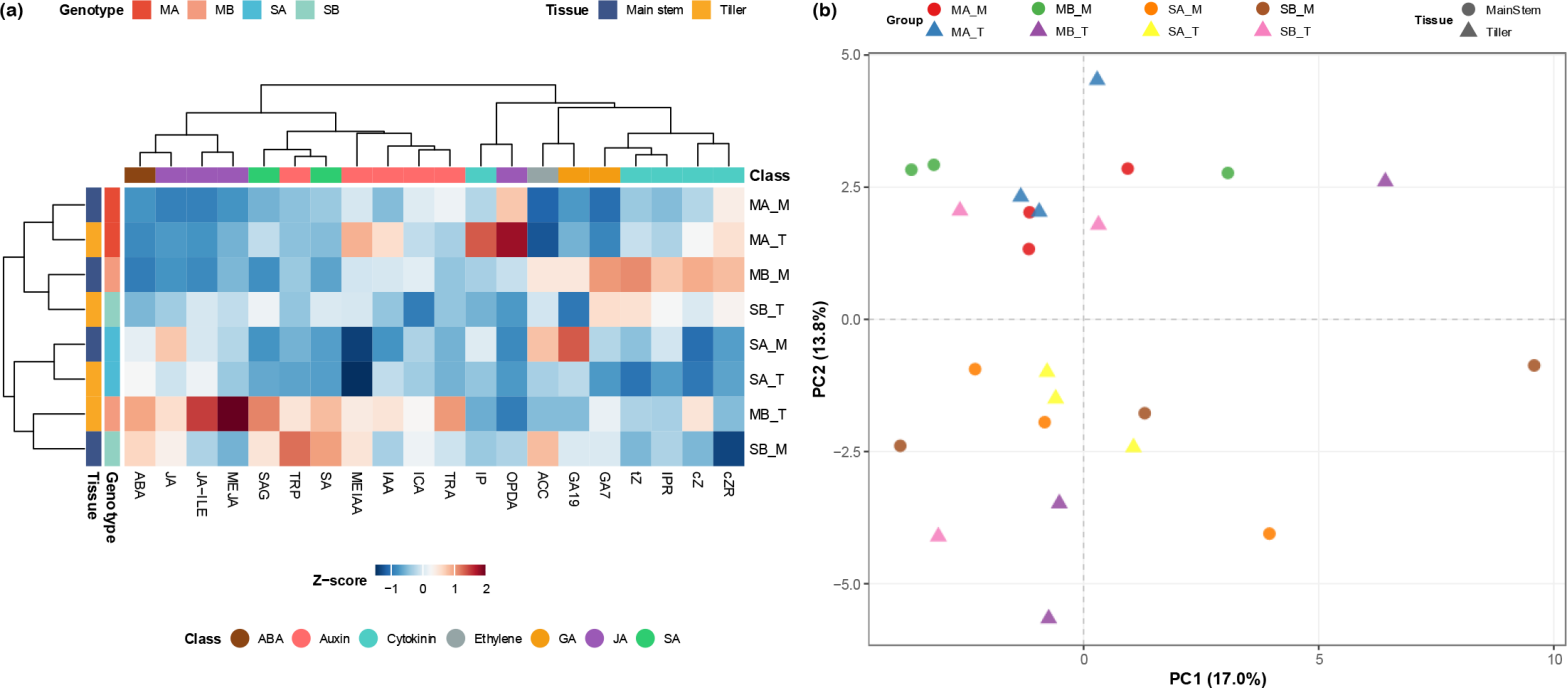
Hormonal variation across tissues and tillering genotypes. **(a)** Representative heatmap of UV-scaled (z-score standardized) mean profiles of retained hormones. For readability, only selected representative contrasts are shown in the main figure; the complete heatmap including all sample groups is provided in **Supplementary Figure 4**. **(b)** Principal component analysis (PCA) of endogenous hormone profiles. Samples were obtained from main stems (M) and tiller stems (T) of high-tillering (MA, MB) and low-tillering (SA, SB) genotypes (n = 3 biological replicates per group). The plot shows the first two principal components, PC1 (17.0%) and PC2 (13.8%), with percentages of explained variance indicated in parentheses on each axis.

To provide an initial overview of hormonal variation, a heatmap of UV-scaled mean abundance profiles was generated. Of the 109 phytohormones assayed in the targeted panel, 67 were detected in at least one sample, and 36 were retained for statistical analysis after excluding compounds with zero values in more than 50% of all samples. **Figure 2a** shows representative hormonal patterns for clarity, whereas the complete heatmap is provided in **Supplementary Figure 4**. The heatmap revealed distinct phytohormone signatures shaped by both genotype and tissue identity.

In tiller tissues, the hormonal profiles of high-tillering genotypes were generally more active and heterogeneous than those of the low-tillering genotypes. The most prominent pattern was observed in MB_T, which showed strong enrichment of jasmonate-related compounds, including Methyl Jasmonate (MEJA) and Jasmonoyl-Isoleucine (JA-ILE), together with elevated levels of several auxin-associated metabolites such as IAA, Tryptamine (TRA), and Methyl Indole-3-acetate (MEIAA). By comparison, MA_T displayed a different but still growth-associated profile, characterized by relatively high 12-oxo-phytodienoic acid (OPDA) and cytokinin-related signals, including cZR, cZ, IP (N6-Δ2-isopentenyl adenine), and iP9G (isopentenyladenine-9-glucoside). In contrast, the tiller tissues of low-tillering genotypes (especially SA_T) showed broadly reduced cytokinin-associated signals together with comparatively weaker auxin- and jasmonate-related enrichment.

In the main stems, the hormonal contrast followed a different pattern. Low-tillering genotypes accumulated higher levels of ACC, ABA, JA (Jasmonic acid), and GA19, together with several indole-related metabolites, whereas high-tillering genotypes were relatively enriched in specific cytokinin species. This cytokinin-associated pattern was especially evident in MB_M, which showed the strongest enrichment of trans-zeatin (tZ), cZR, and cZ, together with elevated GA7. By contrast, SB_M was distinguished by relatively high Indole and cZ9G (cis-Zeatin-9-glucoside) signals.

Tissue comparisons within each tillering background further highlighted contrasting allocation patterns. In the high-tillering background, tiller tissues—particularly MB_T—displayed stronger enrichment of jasmonate- and auxin-related metabolites relative to their corresponding main stems, whereas in the low-tillering background the opposite tendency was more apparent, with main stems retaining higher levels of ACC-, GA19-, and indole-associated metabolites than tiller tissues.

To statistically validate the hormonal clustering patterns, PERMANOVA was performed. Global PERMANOVA confirmed that the experimental groups collectively explained a statistically significant proportion of hormonal variance (R² = 0.348, P = 0.025), with homogeneous within-group dispersions confirmed prior to interpretation (Beta-dispersion test, P = 0.729). However, when variance was partitioned by experimental factor in the two-way PERMANOVA, neither tillering capacity (R² = 0.060, P = 0.056) nor tissue type reached statistical significance, likely reflecting the limited statistical power imposed by the small sample size (n = 3 per group) once variance is distributed across individual factors.

This pattern is highly consistent with the tissue-specific structure observed in the PCA score plot (**Figure 2b**), where the first two principal components capture 30.8% of the total variance. Compared to the highly concentrated variance in the transcriptome (>60%), this broader distribution reflects the multidimensional dispersion characteristic of dynamic downstream hormonal signals. A clear and precise separation was observed between the main stems (M) of high- and low-tillering sugarcane varieties. The main stem samples from high-tillering genotypes (MA_M, MB_M) were consistently located in the lower quadrants (negative PC2 scores), whereas those from low-tillering varieties (SA_M, SB_M) exclusively occupied the upper quadrants. In stark contrast, the tiller stem (T) samples showed a much more complex, scattered distribution across all quadrants, with considerable overlap and no clear trend of separation. This within-group heterogeneity in tiller samples effectively dilutes the global statistical signal. Taken together, these results indicate that while the hormonal composition of the main stem is robustly correlated with tillering capacity, the hormone profiles in tiller stems are highly heterogeneous and do not follow a consistent genotype-dependent pattern.

Supervised OPLS-DA of the hormonal profiles identified a set of discriminative metabolites associated with tillering capacity between high- and low-tillering genotypes (**Supplementary Table 4**). Biological replicates showed acceptable clustering, though inter-sample variance was higher than in the transcriptome data. In the main stems (M), high-tillering varieties established a robust pro-growth environment characterized by a pronounced upregulation of auxin-associated metabolites, most notably TRA, which increased dramatically in the MA genotype (Log_2_FC = 4.97 compared to SA). This was coupled with a consistent and strong suppression of both the ethylene precursor, ACC (MA vs. SA, Log_2_FC = -2.26), and the growth inhibitor ABA (MB vs. SB, Log_2_FC = -1.42). The coordinated promotion of auxin signaling alongside the suppression of key inhibitory pathways represents the most distinct metabolic signature associated with high tillering capacity.

Tissue-specific modulation was also evident. The jasmonate pathway was preferentially activated in the tiller stems (T) of high-tillering genotypes, with significant accumulation of precursors like OPDA (MA vs. SA, Log_2_FC = 1.91). Furthermore, subpopulation-specific differences emerged: cytokinin derivatives, such as 6-benzyladenosine (BAPR), were uniquely elevated in MA tillers, while gibberellin A8 (GA8) predominated in the main stems of the MB genotype. These findings demonstrate that tillering efficiency is associated with a spatiotemporal coordination of multiple hormone pathways, with distinct metabolic adaptations occurring in a tissue- and genotype-specific manner.

Notably, hormonal profiles exhibited greater inter-sample variance than the transcriptomic data, a phenomenon consistent with the complex, multi-level regulation inherent to phytohormone networks.

Despite this variability, the distinct hormonal signature identified in the main stems likely plays a significant role in regulating the differences in tillering capacity among these sugarcane varieties.

### Analysis of ionomics data

**Figure 3:**
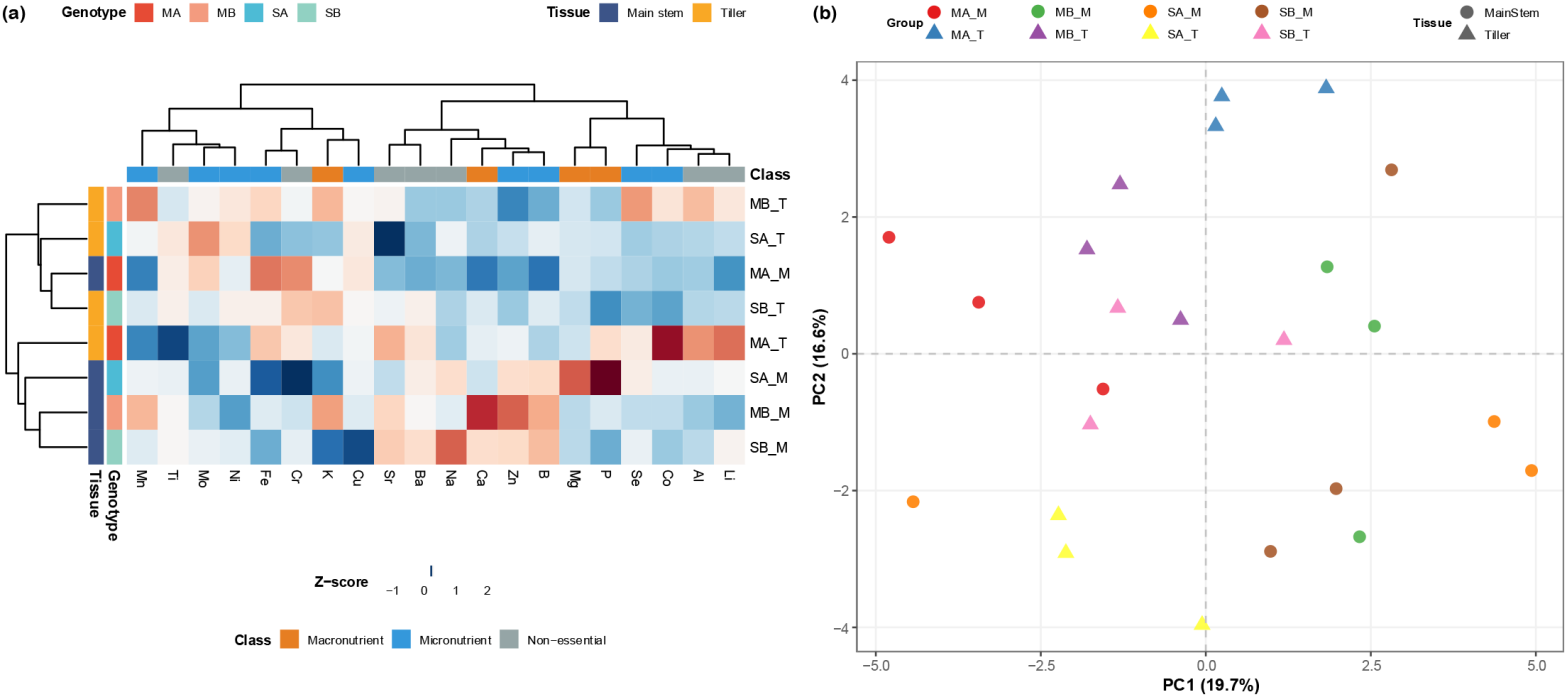
Ionomic variation across tissues and tillering genotypes. **(a)** Representative heatmap of UV-scaled mean elemental profiles, showing 20 elements spanning macronutrients, micronutrients, and non-essential elements. The complete heatmap of all 29 elements and all sample groups is provided in **Supplementary Figure 5**. **(b)** Principal component analysis (PCA) of mineral element (ionomic) profiles. Samples were obtained from main stems (M) and tiller stems (T) of high-tillering (MA, MB) and low-tillering (SA, SB) genotypes (n = 3 biological replicates per group). The plot shows the first two principal components, PC1 (19.7%) and PC2 (16.6%), with percentages of explained variance indicated in parentheses on each axis.

To provide an overview of ionomic variation among samples, a heatmap of UV-scaled mean elemental profiles was generated for all 29 mineral elements measured. **Figure 3a** presents a representative view of 20 elements spanning macronutrients, micronutrients, and non-essential elements, whereas the complete heatmap is provided in **Supplementary Figure 5**. The heatmap revealed distinct ionomic signatures associated with both tissue identity and tillering capacity.

A clear differentiation was observed between main stems and tiller stems. In tiller tissues of the high-tillering genotypes (MA_T and MB_T), relatively higher standardized signals were observed for several micronutrient- and non-essential-element-associated ions, including Co, Li, Se, and Fe, whereas the corresponding low-tillering tiller tissues (SA_T and SB_T) showed relatively higher signals for elements such as Mo and V and comparatively lower values for Co and Se.

In main stems, the ionomic contrast between high- and low-tillering genotypes followed a different pattern. High-tillering genotypes (MA_M and MB_M) showed relatively elevated standardized signals for elements including Fe, K, W, and Cr, whereas low-tillering genotypes (SA_M and SB_M) were comparatively enriched in Na, P, Mg, and B. In addition, MA_M was characterized by particularly high Cr- and Fe-associated signals, while MB_M showed relatively stronger Ca- and Zn-associated enrichment.

Overall, these results indicate that contrasting tillering genotypes differ in tissue-specific elemental distribution patterns rather than showing a uniform shift across the full ionomic spectrum. To further summarize the global structure of this variation, principal component analysis (PCA) was subsequently performed.

To statistically evaluate the global ionomic variation and validate the sample clustering patterns, PERMANOVA was conducted. Global PERMANOVA revealed that the experimental groups collectively explained a highly significant proportion of ionomic variance (R² = 0.490, P = 0.001), with homogeneous within-group dispersions confirmed prior to interpretation (Beta-dispersion test, P = 0.753).

Two-way PERMANOVA identified a significant main effect of tillering capacity (R² = 0.093, P = 0.005) and, critically, a highly significant interaction between tillering capacity and tissue type (R² = 0.103, P = 0.002). This interaction indicates that the pattern of ionomic differentiation between main stem and tiller tissues is fundamentally modulated by tillering phenotype. Consistent with this, the PCA score plot (**Figure 3b**) visually corroborates these genotype-dependent trajectories: the spatial shift from main stems to tillers in high-tillering varieties — such as the pronounced upward displacement of MA_T relative to MA_M along PC2 — follows a completely different directional trajectory compared to low-tillering varieties, where SA_T separates sharply toward the negative PC2 pole. Together, the significant statistical interaction and these divergent spatial patterns provide compelling evidence that tillering capacity dictates a fundamentally distinct redistribution of mineral elements between stem compartments.

To investigate the chemical basis for the distinct clustering observed in the PCA plot, the fold-changes of key differential ions (VIP > 1) were analyzed (**Supplementary Table 5**). The results indicate that high- and low-tillering genotypes exhibit fundamentally different ion accumulation strategies in their tissues, which explains their separation.

In tiller stems (T), high-tillering genotypes (MA_T, MB_T) showed a significant enrichment of key micronutrients compared with their low-tillering counterparts. Most notably, the concentrations of Fe and Ti in MA_T were dramatically elevated relative to SA_T, with Log_2_ fold changes of approximately 3.5 and 4.1, respectively. Similarly, MB_T tillers accumulated significantly more Se and Co than SB_T tillers (Log_2_FC ≈ 1.5 and 1.4, respectively). This indicates that the enrichment of specific micronutrients represents a key ionomic feature supporting the growth and development of tillers in high-tillering varieties.

Meanwhile, a distinct pattern of ion accumulation was observed in the main stems (M). For example, the high-tillering MB_M genotype accumulated significantly more Al, Li, W, and K when compared to the low-tillering SB_M (Log_2_FC > 1.4). In contrast, the MA_M main stems contained lower concentrations of Al and B than the SA_M stems.

In summary, the tillering capacity in sugarcane is strongly linked to its ion acquisition and allocation strategies. High-tillering genotypes are particularly adept at enriching micronutrients such as Fe and Ti in their tillers while making specific ion adjustments in the main stem. These differences form the ionomic basis for the separations observed in the PCA, linking the elemental profiles to the plant’s branching phenotype.

### Multi-omics joint analysis

**Figure 4:**
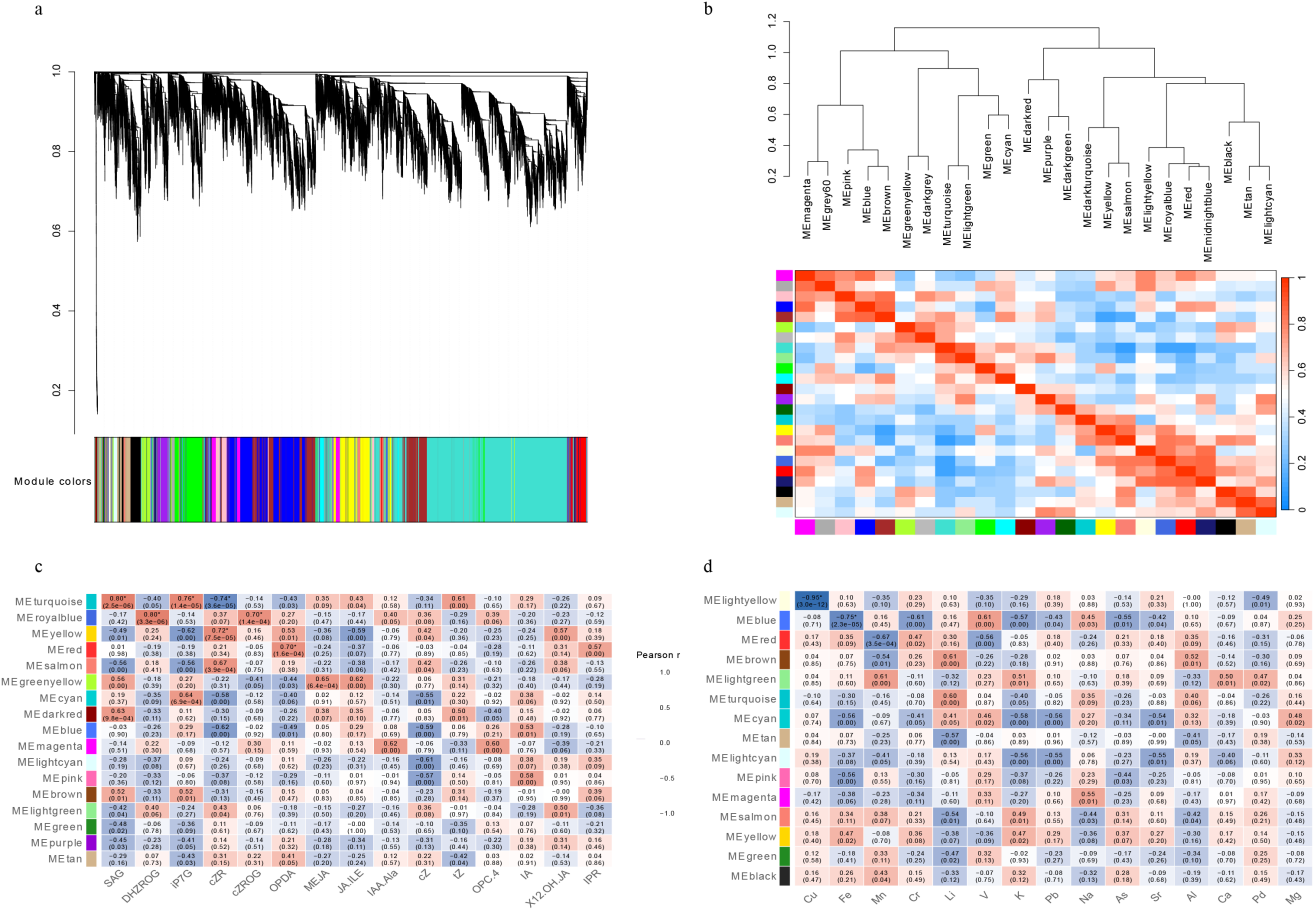
Weighted Gene Co-Expression Network Analysis (WGCNA) of transcriptomic data and module–trait associations. **(a)** Hierarchical clustering dendrogram of filtered transcripts used for weighted gene co-expression network analysis (WGCNA), with the corresponding module color assignment shown below. **(b)** Clustering of module eigengenes and eigengene adjacency heatmap, showing the relationships among co-expression modules at the eigengene level. **(c)** Correlations between module eigengenes and phytohormone traits. **(d)** Correlations between module eigengenes and ionomic traits. In panels **(c)** and **(d)**, each cell displays the Pearson correlation coefficient, with the nominal p value shown in parentheses. Associations supported after Benjamini–Hochberg false discovery rate (FDR) correction are marked with an asterisk (*). For clarity, only modules and traits with at least one nominally significant association (p <no><</no> 0.05) are displayed; the complete correlation matrices for all 22 modules are provided in **Supplementary Figures S6**, **S7**. The soft-thresholding power selection analysis is shown in the **Supplementary Materials**.

Based on WGCNA analysis, genes from the transcriptomic dataset were clustered into 17 co-expression modules, each assigned a color label by the WGCNA algorithm (**Figures 4a, b**). Among these, five color-labeled modules—turquoise (9,008 genes), blue (3,268 genes), brown (2,886 genes), yellow (1,612 genes), and green module—showed significant KEGG pathway enrichment (**Supplementary Table 6**).

Further heatmap and clustering analyses revealed distinct correlation patterns among these key modules (**Figure 4c**). The blue module showed a strong negative correlation with the green module and weaker negative correlations with the yellow and brown modules, while displaying minimal association with the turquoise module. In contrast, the green and yellow modules clustered closely and showed a moderate positive correlation. The turquoise and brown modules formed separate clusters and were negatively correlated with the green and yellow modules.

KEGG enrichment analysis indicated that these five modules were associated with distinct functional categories (**Supplementary Table 6**). The blue module was enriched in pathways related to photosynthesis and circadian rhythm. The green module was enriched in carbon fixation and nitrogen metabolism, whereas the yellow module was enriched in hormone signaling and stress-responsive pathways. In contrast, the turquoise and brown modules were enriched in protein synthesis-related pathways, including ribosome function and aminoacyl-tRNA biosynthesis. Together, these results indicate that the major co-expression modules captured functionally distinct molecular patterns associated with contrasting tillering phenotypes.

To examine module–trait relationships, module eigengene correlations with hormone- and ion-related traits were quantified using Pearson correlation coefficients, and the resulting p values were adjusted for multiple comparisons using the Benjamini–Hochberg false discovery rate (FDR) procedure. The full module–trait correlation matrices are provided in **Supplementary Figures S6**, **S7**, while the main figure (**Figures 4c, d**) displays only associations meeting at least nominal significance (raw p < 0.05), with FDR-supported associations additionally indicated by asterisks. After correction, only a limited subset of module–trait associations remained statistically significant.

Among hormone-related traits, the turquoise module showed significant positive correlations with SAG (r = 0.80, FDR = 0.0017) and iP7G (r = 0.76, FDR = 0.0048), together with a significant negative correlation with cZR (r = −0.74, FDR = 0.0093). The yellow module also retained a significant positive correlation with cZR (r = 0.72, FDR = 0.0155). In addition, the red module showed a significant positive correlation with OPDA (r = 0.70, FDR = 0.0236), whereas the royal blue module showed significant positive correlations with Dihydrozeatin Riboside O-glucoside (DHZROG) (r = 0.80, FDR = 0.0017) and cis-Zeatin Riboside O-glucoside (cZROG) (r = 0.70, FDR = 0.0236).

Among ion-related traits, two module–trait associations remained significant after FDR correction. The lightyellow module showed a strong negative correlation with Cu (r = −0.95, FDR = 1.91 × 10^-9^), whereas the blue module showed a significant negative correlation with Fe (r = −0.75, FDR = 0.0073).

In addition to these FDR-supported associations, several further module–trait correlations met the raw screening criteria used for exploratory WGCNA interpretation (raw p < 0.05 together with substantial correlation magnitude), but did not remain significant after multiple-testing correction. These nominal associations were therefore retained only as hypothesis-generating patterns for candidate prioritization and are reported in **Supplementary Table 7**.

To prioritize candidate genes associated with the most relevant module–trait relationships, key traits were selected using a two-tier strategy: FDR-supported associations were prioritized as the most statistically robust signals, while additional raw p–based module–trait correlations with substantial correlation magnitude were retained as exploratory patterns for candidate generation (**Supplementary Table 7**). Hub genes were then prioritized for each selected module–trait association according to the criteria described in the Methods, and the highest-ranked genes were retained for downstream analysis.

Integration of module–trait associations with hub-gene expression profiles revealed distinct co-expression patterns across genotypes and tissues (**Supplementary Table 7**). The yellow module, which was positively associated with cZR, was enriched in signaling-related functions, and several of its top-ranked hub genes showed higher expression in the main stems of high-tillering genotypes (MA and MB) than in low-tillering genotypes (SA and SB). The turquoise module, enriched in ribosome-related pathways, retained significant associations with SAG, iP7G, and cZR, and many of its top-ranked hub genes were more strongly expressed in low-tillering main stems. The blue module, enriched in photosynthesis-related functions, retained a significant negative association with Fe and contained top-ranked hub genes such as TRINITY_DN122049_c1_g1 and TRINITY_DN116171_c3_g1 that were more highly expressed in SA/SB genotypes.

Overall, the integrated analysis identified a limited set of FDR-supported module–trait associations together with broader exploratory co-expression patterns across hormone and ion datasets. These results provide a network-level framework for subsequent interpretation and candidate-gene prioritization in sugarcane tillering.

## Discussion

### Contrasting transcriptional programs accompany divergent tillering phenotypes

Our integrated multi-omics data support the view that tillering capacity is associated with coordinated shifts across transcriptional and hormonal profiles, rather than reflecting a single quantitative dimension alone. These patterns are broadly consistent with a molecular contrast between growth-associated and defense-/stress-associated processes in the present dataset (Huot et al., 2014; Lozano-Durán and Zipfel, 2015). However, this interpretation should be considered a working hypothesis supported by the present multi-omics patterns rather than a demonstrated resource-allocation mechanism.

Principal coordinate analysis demonstrated that tillering capacity constitutes the dominant axis of transcriptomic divergence (PCo1 = 61.36%), indicating that transcriptomic variation associated with tillering status exceeded that attributable to tissue identity (**Figure 1**). High-tillering genotypes showed more pronounced transcriptional differentiation between main stem and tiller tissues, consistent with greater organ-specific molecular divergence. This greater transcriptomic divergence between source (main stem) and sink (tiller) tissues in high-tillering genotypes suggests that these varieties have established a more complete functional specialization between the two compartments, with each tissue adopting a more distinct molecular identity consistent with its developmental role. Conversely, low-tillering genotypes exhibited more uniform inter-tissue expression patterns, which may reflect reduced tissue differentiation or a less specialized transcriptional organization.

Pathway-level profiles further supported this contrast, revealing stark functional contrasts in the main stem (M) tissues (**Supplementary Table 3**). High-tillering main stems (MA_M, MB_M) showed significant upregulation of pathways related to energy production and cellular metabolism, including Photosynthesis - antenna proteins (ko00196), Carbon fixation (ko00710), and Starch and sucrose metabolism (ko00500). Notably, these high-tillering stems also showed strong enrichment for key secondary metabolism pathways, specifically Phenylpropanoid biosynthesis (ko00940) and Flavonoid biosynthesis (ko00941). These findings are consistent with a growth-associated transcriptional profile characterized by elevated energy metabolism together with enhanced biosynthetic capacity for structural and signaling-related secondary metabolites (Yao et al., 2021). From a source-sink perspective, this transcriptional state positions the main stem of high-tillering genotypes as an active carbon source, with enhanced capacity to fix, metabolize, and potentially export photosynthates to support tiller development.

In contrast, the transcriptomic profile of low-tillering main stems was distinct, characterized by strong enrichment of pathways related to protein production machinery: Ribosome (ko03010), Ribosome biogenesis in eukaryotes (ko03008), and Aminoacyl-tRNA biosynthesis (ko00970). Although ribosome activity is often associated with active growth, increased translational capacity can also occur during stress or defense responses that require rapid synthesis of regulatory proteins. The main stem of low-tillering genotypes showed a transcriptional profile enriched in protein synthesis-related pathways, suggesting a distinct regulatory state relative to high-tillering genotypes, although the physiological consequences of this enrichment remain to be clarified (Weis et al., 2015). While defense-related pathways, such as Plant-pathogen interaction (ko04626), were enriched in low-tillering genotypes, they were specifically in the tiller (T) tissues (e.g., in MA_T vs SA_T and MA_T vs SB_T comparisons). Critically, the activation of defense-related pathways in low-tillering tiller tissues, rather than reflecting an active immune response, may be consistent with altered source–sink coordination, although direct evidence for carbon limitation was not assessed in this study. Taken together, high-tillering stems were enriched in energy and secondary metabolic pathways, whereas low-tillering stems were enriched in protein synthesis-related pathways—a functional divergence consistent with distinct physiological states between the two tillering groups (Campos et al., 2016; Maeda et al., 2023).

These contrasting tissue-specific patterns—translational enrichment in the main stems and defense activation in the tillers—provide a unified molecular context for the low-growth phenotype. Given that all genotypes were grown under identical, non-stressed field conditions, this functional divergence may reflect a constitutive, defense-associated transcriptional state, although this interpretation remains hypothetical. Under the Optimal Defense hypothesis, slower-growing phenotypes tend to maintain higher levels of preformed defenses than fast-growing counterparts (G. A. Rosenthal, 1992; Kempel et al., 2011). Sustaining such a baseline “stress-prepared” state demands continuous protein turnover, and plant immunity is known to rely on translational reprogramming—including upregulation of ribosome biogenesis—to prioritize the synthesis of defense-related proteins (Delteil et al., 2012; Xu et al., 2017b). Therefore, ribosomal enrichment in low-tillering genotypes may reflect increased translational demand associated with defense-related processes rather than enhanced growth per se. Thus, the enriched ribosomal pathways in low-tillering main stems may reflect altered translational demand associated with this transcriptional state; however, whether this contributes directly to reduced axillary bud outgrowth remains unresolved. Together, these findings suggest a working hypothesis in which the degree of transcriptional specialization between source and sink tissues may itself be a determinant of tillering efficiency: high-tillering genotypes achieve a clear functional division of labor between main stem and tiller, whereas low-tillering genotypes fail to establish this division, with both tissues becoming occupied by defense- and protein synthesis-associated transcriptional demands that may be associated with reduced tiller outgrowth.

### Hormonal crosstalk is associated with key gene network states

While DEG analysis highlighted significant fold changes in specific hormones, including the auxin precursor TRA, WGCNA further indicated that module-level transcriptomic variation was associated with multiple hormone-related traits, including cytokinins (CKs), jasmonate-related compounds, and salicylate-associated metabolites. Notably, the hormonal profiles displayed substantial variability across genotypes and tissues. Such variability is not unexpected because plant hormones function as highly dynamic signaling molecules whose concentrations are strongly influenced by tissue identity, developmental stage, and local metabolic activity.

Among the hormones examined, cZR showed some of the strongest FDR-supported module-level associations, with opposing correlation patterns across modules that differed in their genotypic enrichment. These contrasting associations may partly reflect spatially differentiated hormonal regulation between the main stem and tiller tissues, which represent physiologically distinct compartments involved in systemic signaling and local bud development, respectively. The turquoise module—a module enriched in protein synthesis-related functions and strongly associated with low-tillering main stems—exhibited a significant negative correlation with cZR (r = −0.74, FDR = 0.0093), indicating that higher cZR levels were associated with lower eigengene values of this module. Such tissue-dependent hormone–network relationships suggest that the regulatory role of cytokinins may differ between developmental contexts, potentially contributing to the contrasting growth programs observed in high- and low-tillering genotypes. Conversely, the yellow module—associated with hormone signaling and stress-responsive pathways—showed a significant positive correlation with cZR (r = 0.72, FDR = 0.0155). Analysis of the top 20 hub genes for these associations (**Supplementary Table 7**) supported these patterns at the gene level: turquoise–cZR hub genes were consistently more highly expressed in low-tillering main stems (SA, SB), whereas yellow–cZR hub genes showed the opposite trend, with higher expression in high-tillering genotypes (MA, MB). These opposing associations suggest that cZR levels are among the strongest hormonal correlates of the major transcriptional states captured by WGCNA, consistent with the reported involvement of cytokinin signaling in axillary bud regulation (Luo Mingzhu et al., 2002; Argueso et al., 2012; Qi et al., 2025). Taken together, these opposing module--cZR associations suggest that cZR may function as a molecular switch associated with two contrasting transcriptional states: a growth-oriented state characterized by active hormonal signaling (yellow module) and a defense-oriented state characterized by translational enrichment (turquoise module). Whether cZR acts as an upstream regulator or a downstream readout of these transcriptional states remains to be determined, but its consistent association across both module- and gene-level analyses positions it as a priority candidate for future functional investigation. Taken together, these observations indicate that hormonal regulation of tillering is spatially structured, and the balance among multiple hormones rather than the absolute abundance of any single hormone may represent a key determinant of tiller development.

The turquoise module also showed FDR-supported positive correlations with SAG (r = 0.80, FDR = 0.0017) and iP7G (r = 0.76, FDR = 0.0048), both of which are cytokinin-related metabolites. Together with its negative association with cZR, these results suggest that the turquoise module was associated with a specific cytokinin signature in which SAG and iP7G levels were elevated while cZR levels were reduced, a pattern predominantly observed in low-tillering main stems. This cytokinin signature-–elevated inactive or conjugated forms (SAG, iP7G) alongside reduced active cZR-–may reflect a shift toward cytokinin inactivation in low-tillering main stems, consistent with a transcriptional state that prioritizes defense and protein synthesis over growth-promoting hormonal signaling (Argueso et al., 2012; Duan et al., 2019).

Beyond cytokinins, the red module showed a significant positive association with OPDA (r = 0.70, FDR = 0.0236), and the royal blue module showed significant associations with DHZROG (r = 0.80, FDR = 0.0017) and cZROG (r = 0.70, FDR = 0.0236), indicating additional hormone–network associations that remained significant after multiple-testing correction.

In contrast to the FDR-supported cytokinin-related associations described above, several additional hormone–module relationships were observed only at the nominal level (raw p < 0.05) but did not survive FDR correction. These include associations between the turquoise module and ACC (r = 0.77, raw p < 1 × 10^-5^), GA19 (r = 0.77, raw p < 1 × 10^-5^), and SA (r = 0.79, raw p < 1 × 10^-5^). Although these associations did not remain significant after FDR correction, their raw-correlation effect sizes were relatively large, and they are therefore retained here only as exploratory patterns. The suppression of ACC (Log2FC = −2.26 in MA vs. SA) identified in DEG analysis, combined with the nominal turquoise–ACC association, may be consistent with a possible link between ethylene precursor abundance and the transcriptional state enriched in low-tillering genotypes—consistent with reports that excessive ethylene levels can be growth-inhibitory (Vasantha and Kumar, 2017; Cao et al., 2023)—but this interpretation remains tentative pending replication. In contrast, ABA showed weaker and non-significant module-level associations overall, consistent with its more context-dependent roles in bud activity (Patil et al., 2022; Begum et al., 2022).

Beyond the WGCNA-level associations, the tissue-specific hormonal data suggest a possible inter-tissue hormonal coordination mechanism in high-tillering genotypes. The pronounced accumulation of the auxin precursor TRA in high-tillering main stems, concurrent with elevated cZR in their tiller tissues, is consistent with a scenario in which main stem-derived auxin signaling and tiller-localized cytokinin activity operate in a coordinated manner to promote tiller outgrowth. While the auxin-cytokinin interaction in axillary bud regulation is well-established in model grasses such as rice (Duan et al., 2019; Qi et al., 2025), direct evidence for inter-tissue hormonal relay between main stem and tiller compartments was not obtained in this study. Nevertheless, the concurrent hormonal patterns observed here are consistent with such a mechanism and warrant further investigation through tissue-specific hormone transport assays.

### Ionomic variation is associated with stress- and nutrient-related molecular signatures

In parallel with transcriptional and hormonal differentiation, ionomic status emerged as an additional layer associated with variation in tillering capacity. Supported by the significant multivariate interaction between genotype and tissue, the spatial distribution patterns of mineral elements between source and sink tissues appear to be modulated by the tillering phenotype. Unlike transcriptional and hormonal signals, which require active inter-tissue communication between main stem and tiller compartments, mineral element acquisition in established tillers is largely mediated by their own root systems, rendering each tissue capable of independent ionic uptake. The ionomic differences observed between genotypes may therefore reflect genotype-specific differences in overall mineral acquisition capacity and metabolic demand, rather than inter-tissue redistribution per se. In this sense, ionomic variation may represent a downstream metabolic consequence of the transcriptional and hormonal states that characterize contrasting tillering phenotypes, rather than a primary regulatory driver. Furthermore, WGCNA linked ionomic variation with module-level transcriptional patterns, including associations potentially related to stress- and nutrient-associated processes.

Among these signatures, Li, a non-essential element with known cellular effects, showed positive correlations with the defense-associated modules MEturquoise (r = 0.60, raw p = 0.0018) and MEbrown (r = 0.61, raw p = 0.0015). These associations did not survive FDR correction and are therefore interpreted as exploratory patterns; nevertheless, the effect sizes suggest a possible association between the low-tillering transcriptional state and Li-related ionomic variation, although the physiological significance of this relationship remains to be determined.

In contrast, the blue module (photosynthesis-related) exhibited an FDR-supported negative correlation with Fe (r = −0.75, FDR = 0.0073). A nominally significant negative correlation with K (r = −0.57, raw p = 0.0039) was also observed, though this did not survive correction. Taken together, these results suggest an ionomic contrast in which Fe-related variation was robustly associated with the photosynthesis-related blue module, whereas Li-related variation showed exploratory association with defense-associated modules. This pattern is consistent with differential ionomic organization between genotypes, although its physiological basis remains to be clarified. The link between Li and K is consistent with reports of Li–K interaction in plant ion uptake (Hawrylak-Nowak et al., 2012). Although the underlying physiological basis in sugarcane requires further investigation, Li^+^ has been reported to interfere with cation-dependent enzyme activities due to its similar ionic radius (Shahzad et al., 2016) and to inhibit inositol-phosphatase pathways that modulate Ca²^+^ signaling (Jia et al., 2019). These reported mechanisms provide a possible physiological context for the associations observed here, but were not directly tested in the present study.

In addition, the lightyellow module showed a remarkably strong negative correlation with Cu (r = −0.95, FDR = 1.91 × 10^-9^), representing the most statistically robust module–trait association identified across both datasets. While the functional significance of this finding is not yet clear, the strength of this association suggests that Cu status may be tightly linked to the corresponding module eigengene pattern. Notably, Cu serves as a cofactor for ACC oxidase, the enzyme responsible for converting ACC to ethylene. The observation that high-tillering genotypes maintained systemically higher Cu levels alongside significantly reduced ACC (Log2FC = −2.26 in MA vs. SA) raises the possibility that ethylene biosynthesis may be actively suppressed despite sufficient cofactor availability, consistent with a growth-promoting hormonal environment in high-tillering genotypes. However, this interpretation is speculative and would require direct measurement of ACC oxidase activity and ethylene production to substantiate.

This ionomic pattern was also reflected in hub-gene expression. Genes from the MEblue–Fe top 20 list showed distinct expression differences between high- and low-tillering genotypes in main stems, consistent with an association between Fe status and module organization. Previous work has suggested that imbalances in key macronutrients may influence tillering capacity (Yan et al., 2021), and our findings are consistent with this concept.

Importantly, all genotypes were sampled from the same nursery garden under shared management, reducing environmental heterogeneity and strengthening the interpretation that the observed ionomic and transcriptional differences were more likely associated with genotypic differences than with major environmental heterogeneity (de Villemereuil et al., 2016). While we do not infer direct causality, the observed ionomic shifts—particularly the FDR-supported association with Fe and the exploratory patterns involving Li and K—are suggestive of genotype-specific differences in ionomic organization and balance.

A key finding from the ionomic analysis is that Fe and Cu showed systemically higher levels in high-tillering genotypes across both main stem and tiller tissues, rather than exhibiting consistent directional gradients between the two compartments. This pattern suggests that the ionomic advantage of high-tillering genotypes lies in their overall capacity to acquire and maintain higher levels of these essential micronutrients, rather than in their ability to redistribute elements between tissues. Fe plays essential roles in photosynthesis and respiration (Dhaliwal et al., 2022; Gupta et al., 2024), and its systemically elevated levels in high-tillering genotypes are consistent with their transcriptional enrichment of photosynthesis- and carbon metabolism-related pathways, providing further cross-omics support for a more active energy metabolism state in these genotypes. Additional essential nutrient pools (e.g., Ca, Zn, Cu, Mn) also exhibited modest shifts (Engels et al., 2012; Hepler, 2016), suggesting that additional elements may also contribute to the broader physiological context associated with tiller development.

Collectively, these observations indicate that ionomic balance and micronutrient allocation differed systematically between genotypes with contrasting tillering capacities. Rather than acting as primary regulators of tillering, the ionomic differences observed here are more consistently interpreted as downstream reflections of the distinct metabolic states associated with high- and low-tillering phenotypes — states that are more proximally shaped by transcriptional and hormonal regulatory networks. While causal mechanisms remain to be experimentally verified, our data reveal coordinated ionomic–transcriptional patterns associated with contrasting tillering capacities in sugarcane.

### Limitations and future perspectives

This study provides a multi-omic perspective on the molecular basis of contrasting tillering capacities in sugarcane, highlighting distinct transcriptional and metabolic specialization between main stem and tiller tissues. However, several limitations should be noted. First, WGCNA is inherently correlation-based and does not establish direct causal relationships; accordingly, module–trait patterns that did not remain significant after multiple-testing correction should be regarded as exploratory. Second, the present sampling represents a single developmental snapshot at the peak tillering stage; time-series analyses will be required to resolve the temporal progression of these regulatory networks more fully. Third, because sugarcane possesses a highly polyploid genome with extensive allele redundancy and homoeologous gene copies, homoeolog expression bias and transcript fragmentation may influence transcript-level DEG detection and may also affect the estimation of co-expression relationships used for network construction and interpretation. Fourth, the hub genes identified here should be regarded as priority candidates requiring functional validation; although direct genetic manipulation in sugarcane remains challenging owing to its complex polyploid genome, complementary approaches such as transient expression assays or validation in tractable model systems may provide important initial evidence. Finally, whether the divergent molecular profiles observed here extend to differential rhizosphere interactions remains an open question worthy of future investigation.

## Conclusion

In conclusion, our study suggests that sugarcane tillering is associated with coordinated variation across growth-related and defense-/stress-related molecular features. High-tillering genotypes were characterized by cytokinin-related hormonal signatures, higher expression of specific growth- and signaling-associated co-expression modules, and relatively greater micronutrient enrichment in developing tillers, whereas low-tillering genotypes were more strongly associated with stress-related hormonal signatures and defense- and protein synthesis-associated transcriptional modules. Together, these findings are broadly consistent with a growth–defense trade-off interpretation at the molecular level, although direct causal relationships remain to be established.

These findings provide a framework for exploring how productivity and stress resilience might be balanced in future sugarcane breeding efforts. The hub genes identified in key co-expression modules represent priority candidates for future functional validation and, if confirmed, may inform molecular breeding strategies aimed at improving tillering capacity in sugarcane.

## Data Availability

The original contributions presented in the study are included in the article/**Supplementary Material**. Further inquiries can be directed to the corresponding authors.
