# Supplementary material for: Integrated multi-omics analysis reveals hormonal and nutrient networks regulating sugarcane tillering
Source: Front Plant Sci. 2026 Apr 20;17:1755625. doi: 10.3389/fpls.2026.1755625 (PMC13136100; doi:10.3389/fpls.2026.1755625)

Supplementary: Full Module–Mineral Element Relationships

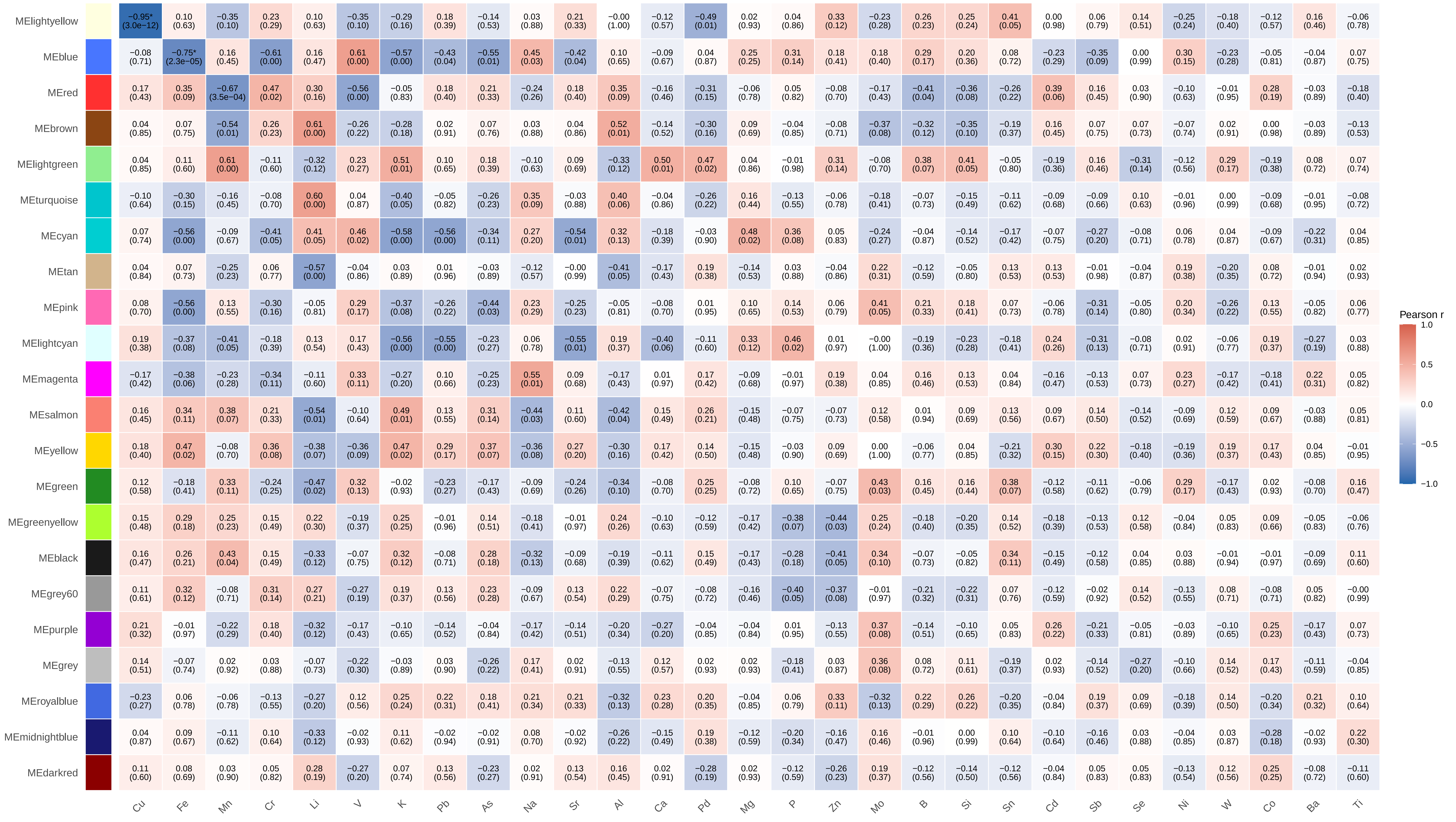

Supplement: Supplementary file 1 [file DataSheet1.zip › Supplement/Figure S7 Full module–ionomic trait correlation matrix.pdf]
