# Supplementary figures and images for: Integrated multi-omics analysis reveals hormonal and nutrient networks regulating sugarcane tillering
Source: Front Plant Sci. 2026 Apr 20;17:1755625. doi: 10.3389/fpls.2026.1755625 (PMC13136100; doi:10.3389/fpls.2026.1755625)

# Scale independence

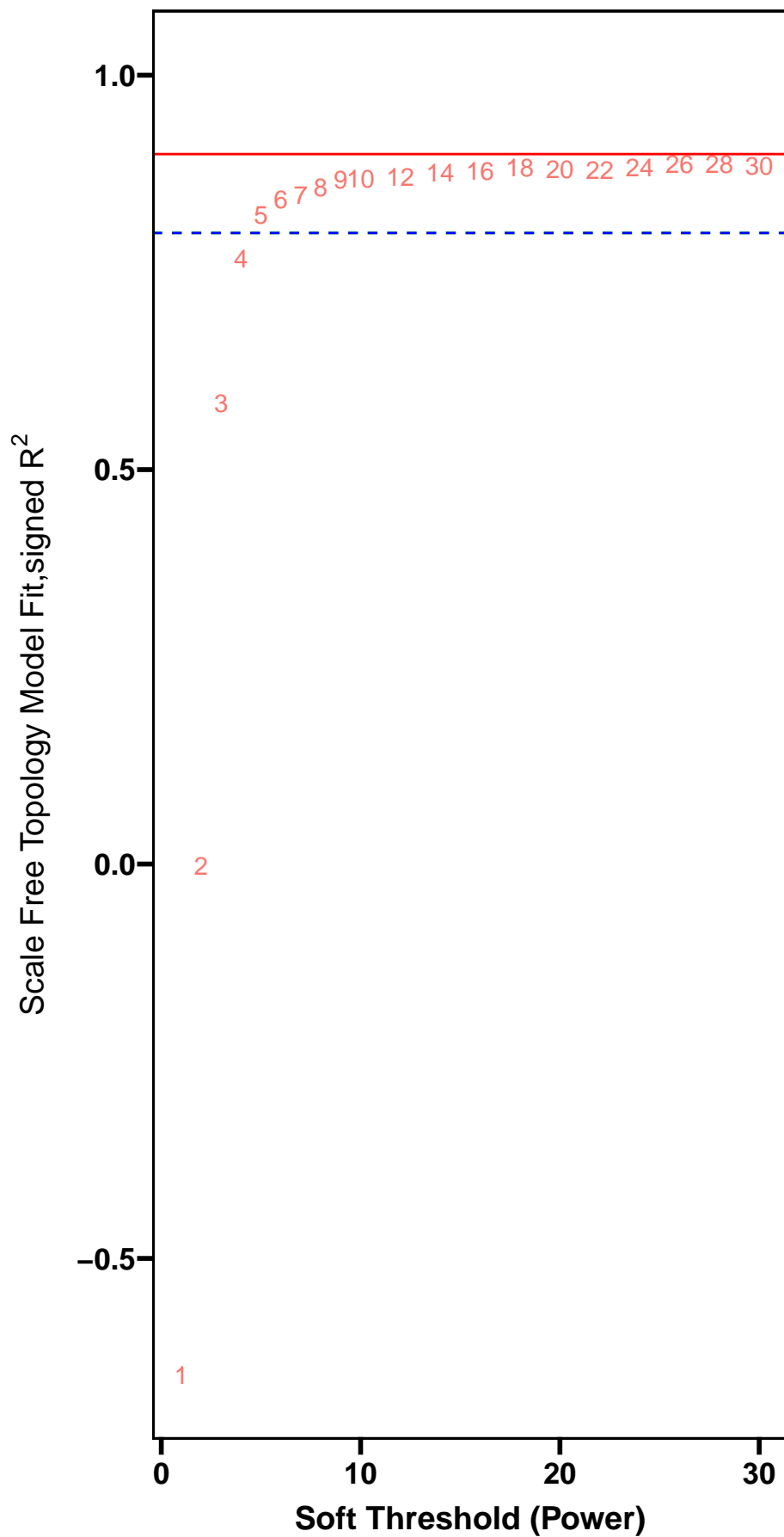

# Mean Connectivity

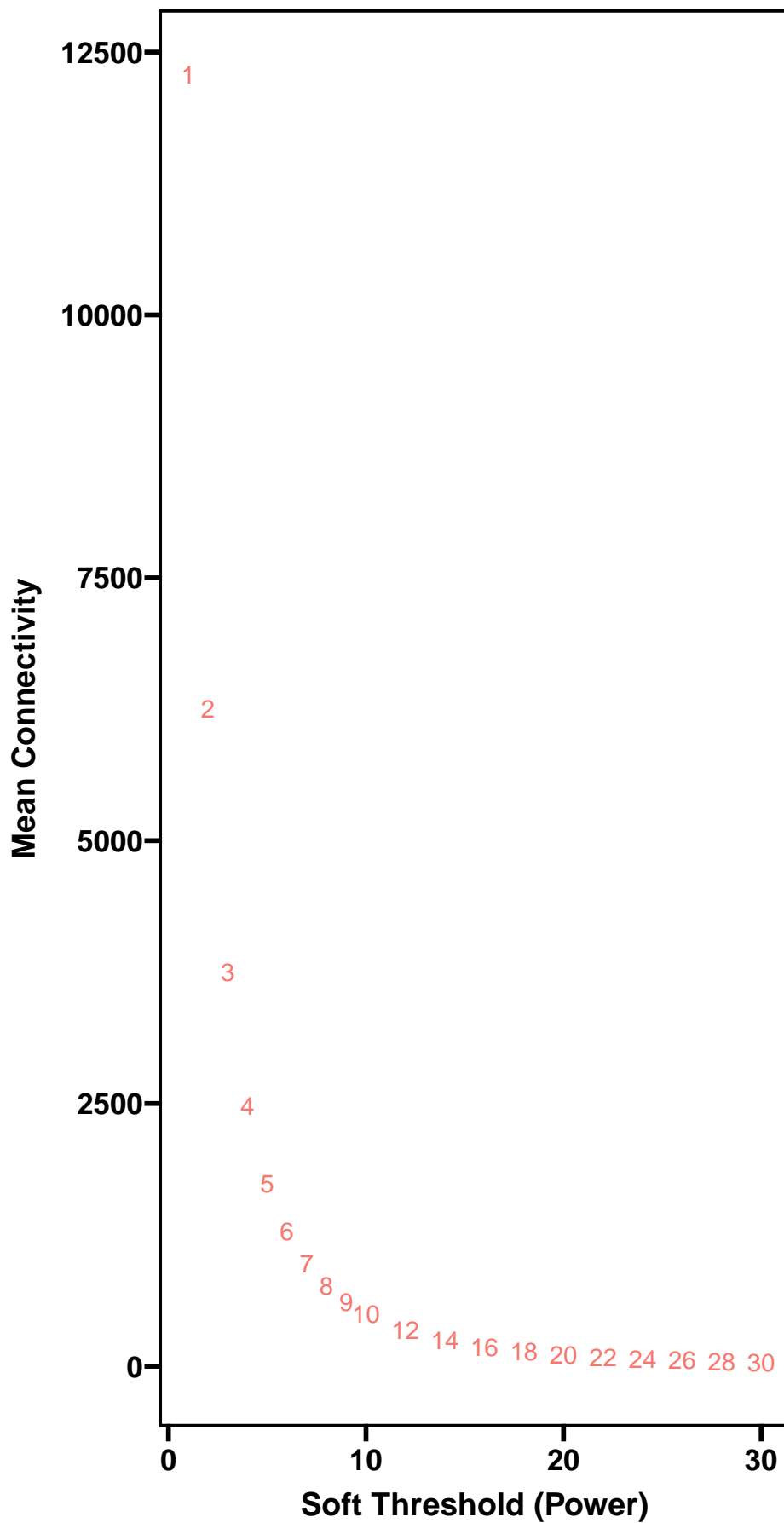

Supplement: Supplementary file 1 [file DataSheet1.zip › Supplement/Figure S1 Scale-free topology fit index across soft-thresholding powers.pdf]

soft connectivity (power = 12 )

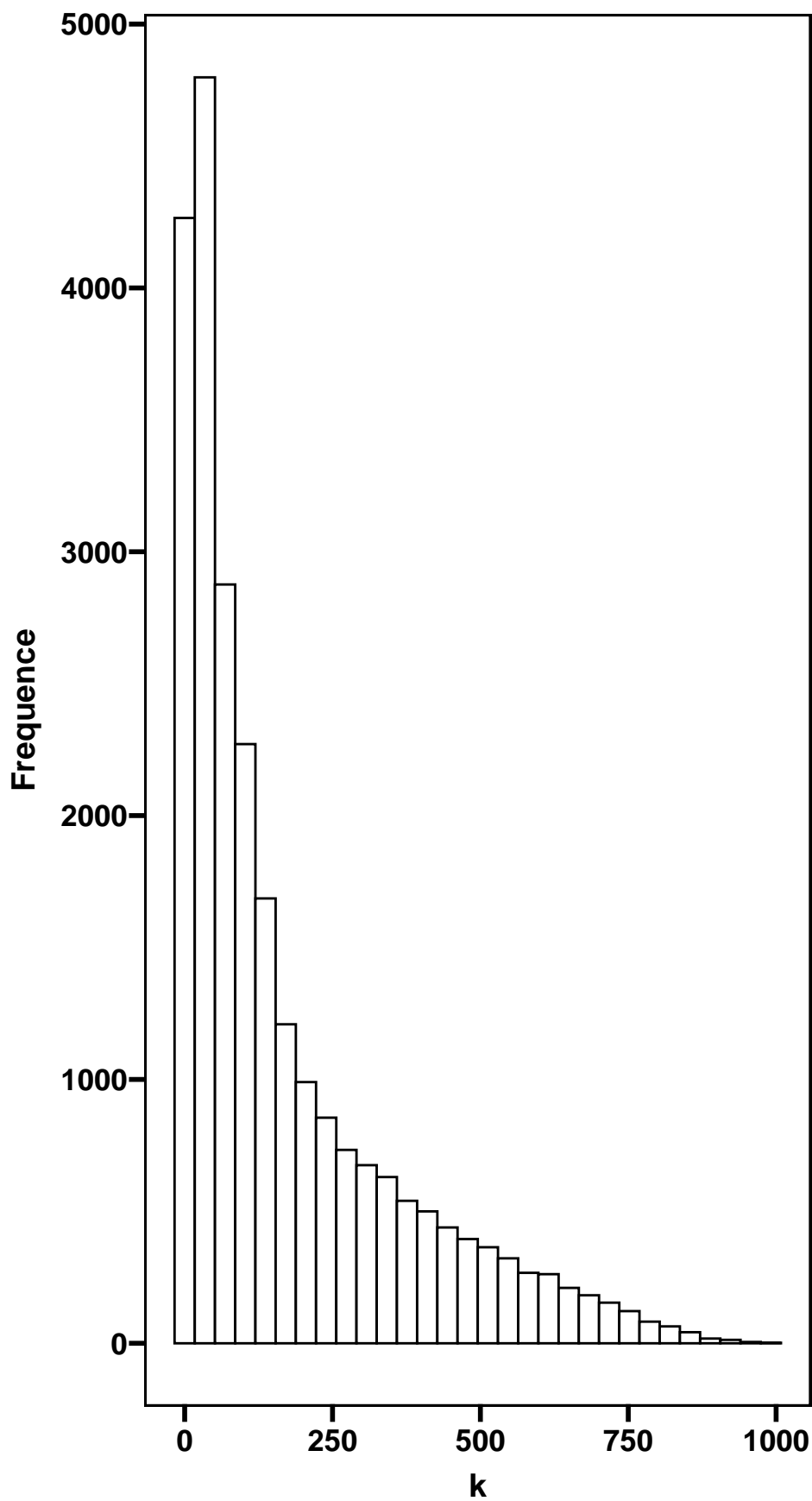

Check Scale free topology  
scale  $R^2 = 0.71$  , slope =  $-1.54$

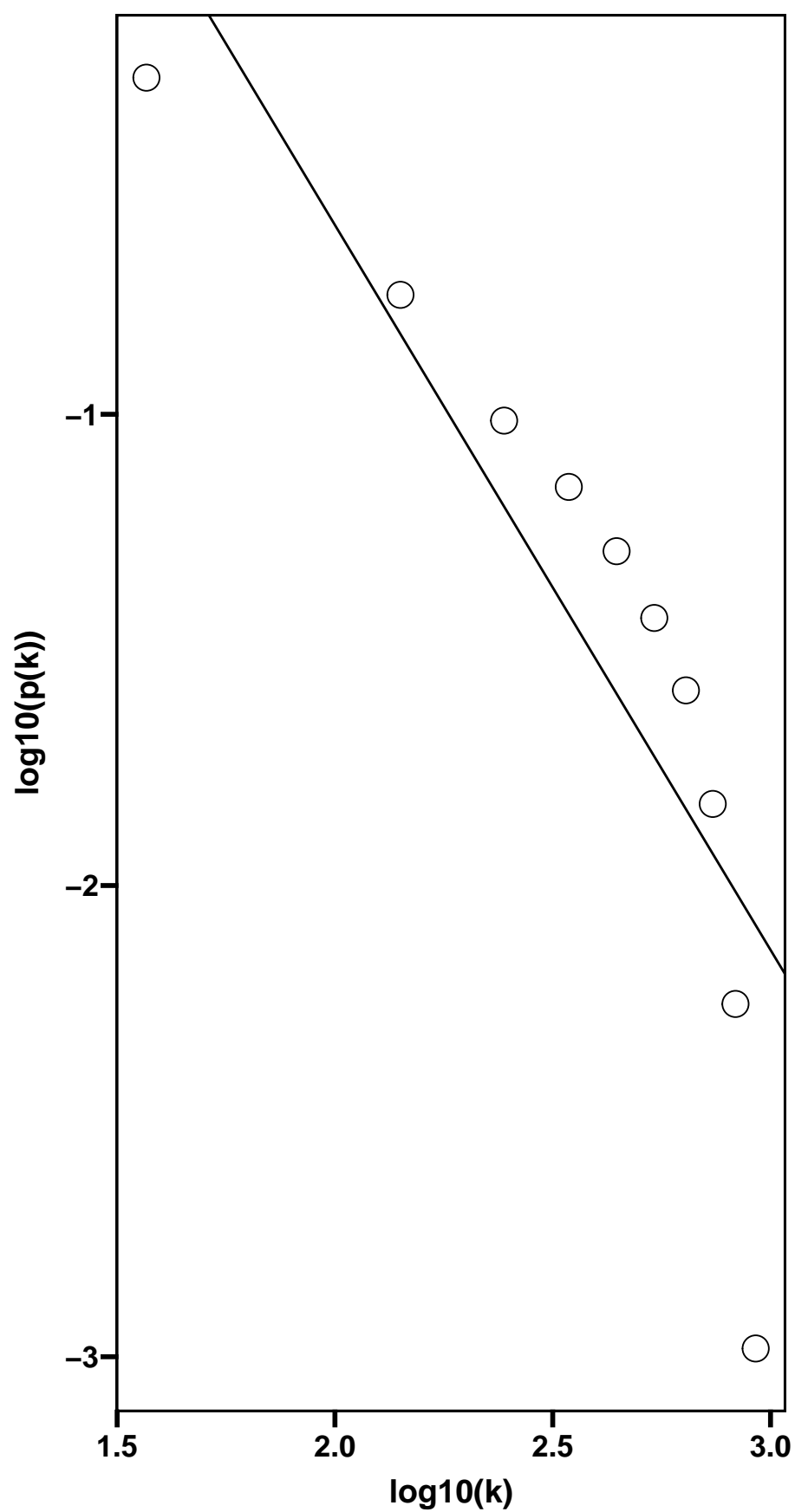

Supplement: Supplementary file 1 [file DataSheet1.zip › Supplement/Figure S2 Mean connectivity across soft-thresholding powers.pdf]

**Hormone Profile Heatmap (UV-scaled, group mean)**

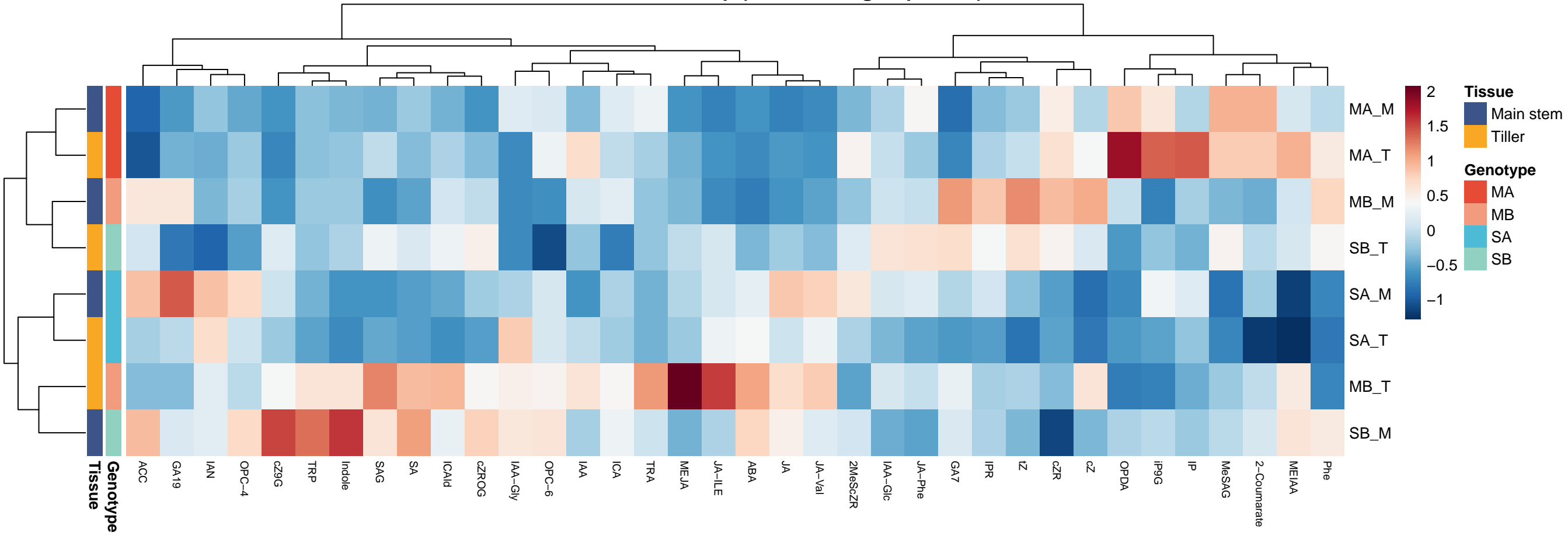

Supplement: Supplementary file 1 [file DataSheet1.zip › Supplement/Figure S4 Heatmap of hormones in sugarcane tissues and genotypes with contrasting tillering capacities.pdf]

Ionic Profile Heatmap (UV-scaled, group mean)

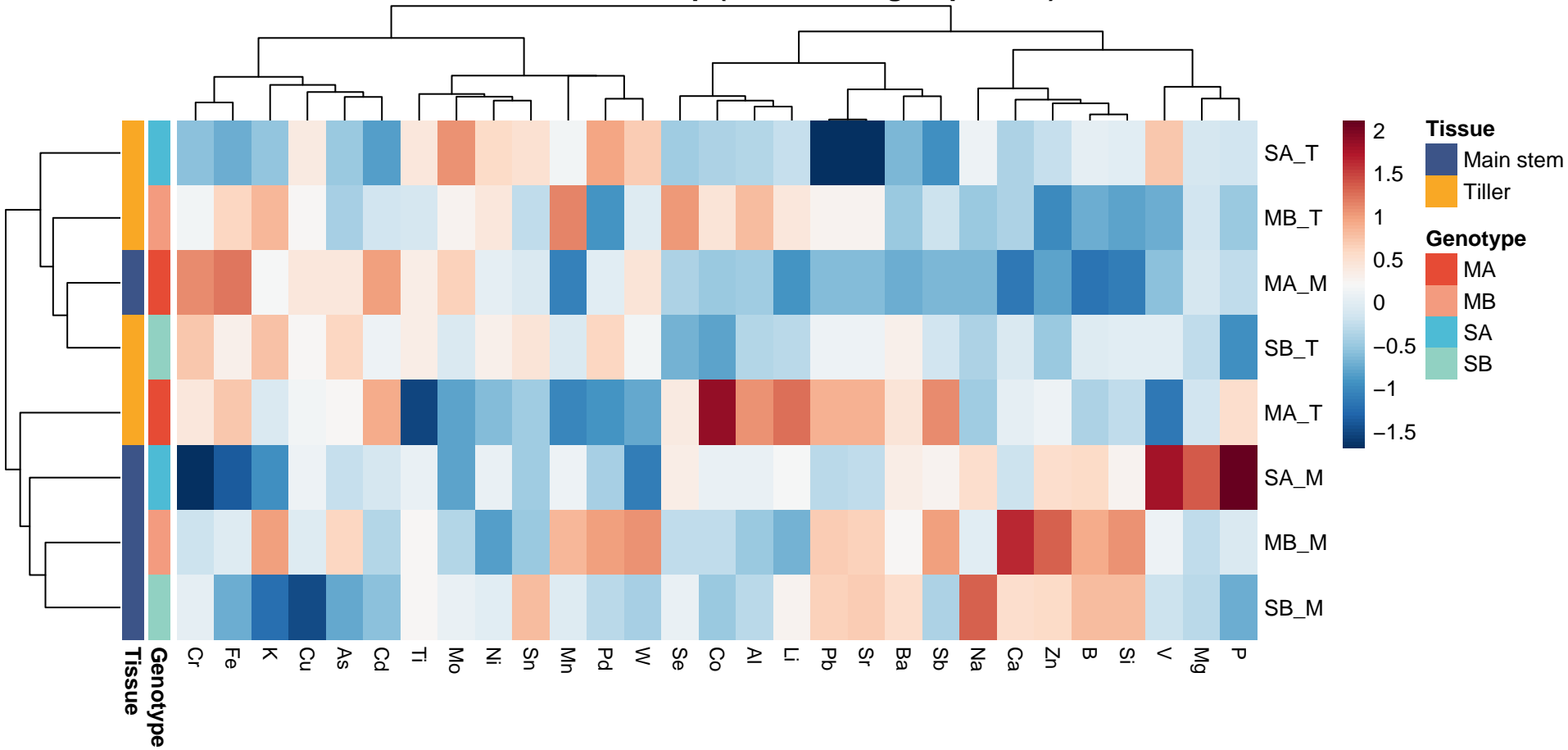

Supplement: Supplementary file 1 [file DataSheet1.zip › Supplement/Figure S5 Heatmap of 29 mineral elements in sugarcane tissues and genotypes with contrasting tillering capacities.pdf]

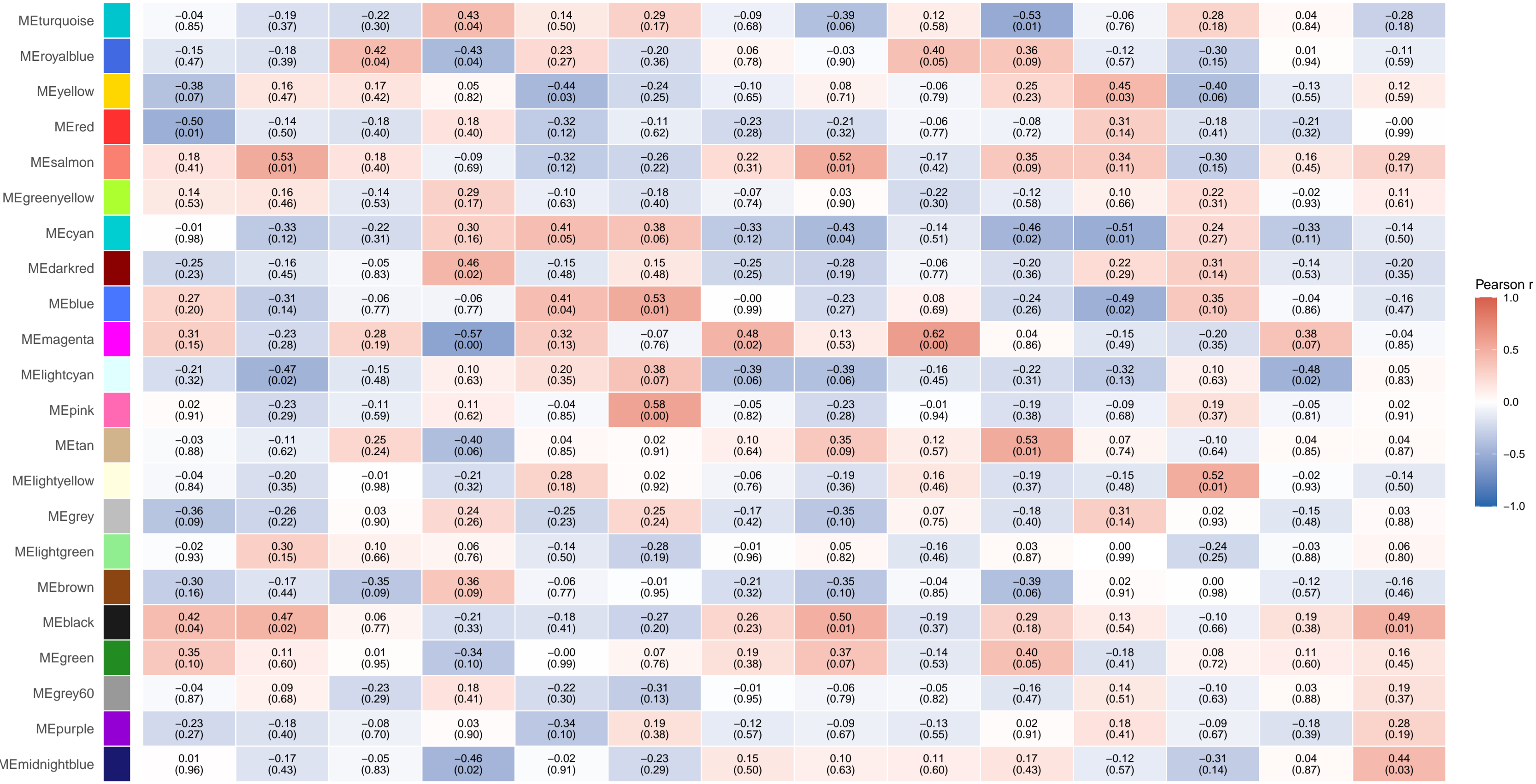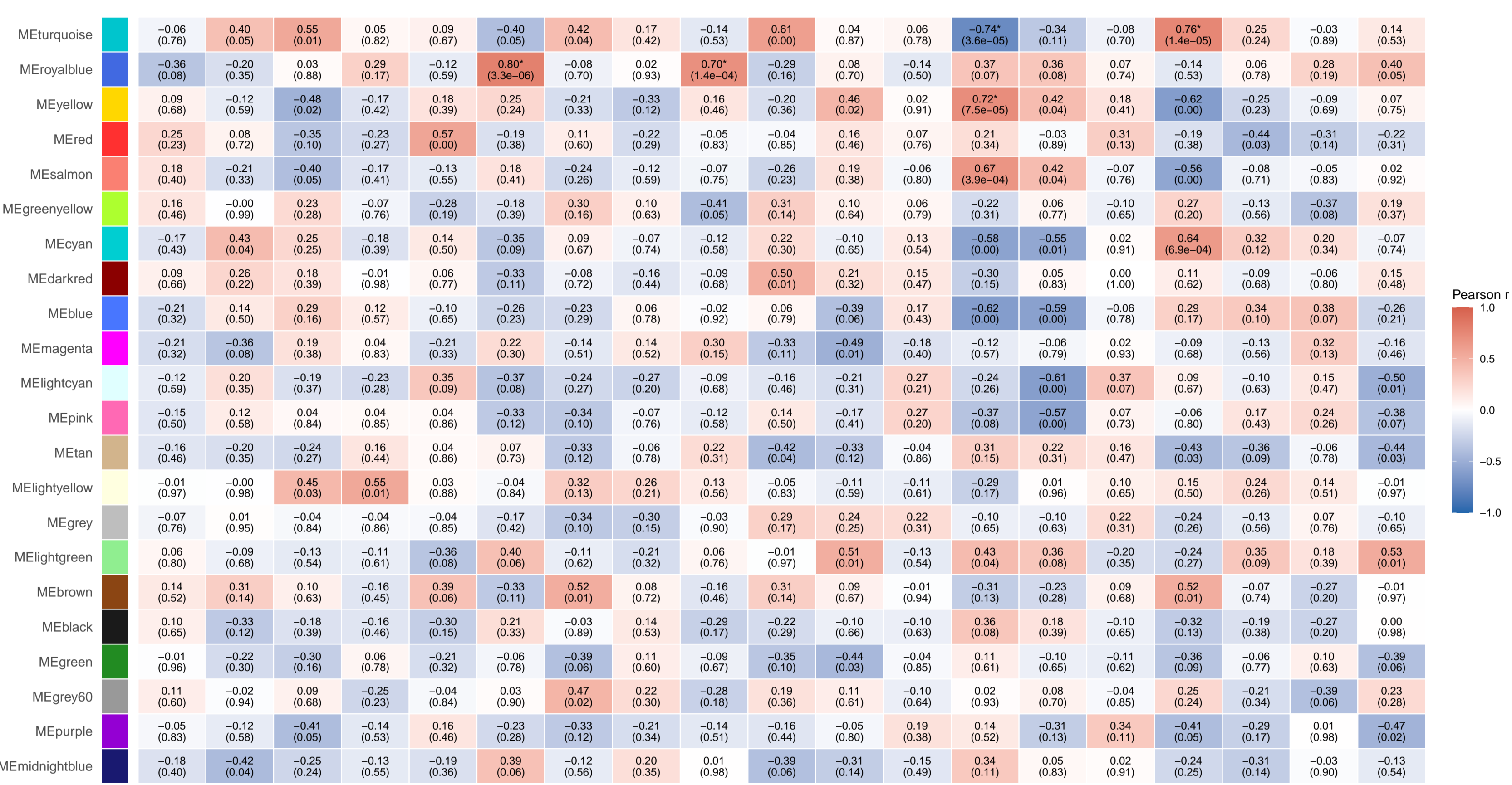

Supplement: Supplementary file 1 [file DataSheet1.zip › Supplement/Figure S6 Full module–hormones trait correlation matrix.pdf]
